# Integrated Multi-Omics and Spatial Transcriptomics Reveal GUK1 as a Prognostic Biomarker Regulated by the TP53-HSF1 Axis in Breast Cancer

**DOI:** 10.32604/or.2026.078813

**Published:** 2026-07-16

**Authors:** Wei Lee, Hung-Yu Lin, Pei-Yi Chu

**Affiliations:** 1 Department of Post-Baccalaureate Medicine, College of Medicine, National Chung Hsing University, Taichung, Taiwan; 2 Research Assistant Center, Show Chwan Memorial Hospital, Changhua, Taiwan; 3 Department of Pathology, Show Chwan Memorial Hospital, Changhua, Taiwan

**Keywords:** Breast cancer, guanylate kinase 1, tumor immune microenvironment, TP53, heat shock factor 1

## Abstract

**Background:** Guanylate kinase 1 (GUK1) is crucial for nucleotide metabolism, yet its impact on breast cancer (BC) progression remains poorly defined. The objective of the present study is to investigate GUK1 as a prognostic biomarker and therapeutic target. **Methods:** We employed a multi-omics approach integrating The Cancer Genome Atlas (TCGA) data, machine learning algorithm, High-Definition spatial transcriptomics (Visium HD), single-cell profiling, molecular docking and experimental validation including *in vitro* knockdown models and Surface Plasmon Resonance (SPR). **Results:** LASSO regression identified *GUK1* as a key metabolic driver. High expression correlated significantly with poor survival and was most pronounced in Human Epidermal Growth Factor Receptor 2 (HER2)-positive and triple-negative subtypes. Spatial transcriptomics revealed *GUK1* strongly colocalizes with expanding cancer cell nests, intensifying with disease stage. Single-cell analysis linked *GUK1* overexpression to an immunosuppressive microenvironment enriched in exhausted T-cells. Clinically and molecularly, *TP53* mutations are highly associated with *HSF1* promoter hypomethylation and subsequent HSF1-mediated GUK1 upregulation. We experimentally confirmed this axis, showing that *HSF1* or *GUK1* knockdown significantly impaired cell migration and suppressed mTOR signaling. Furthermore, while high *GUK1* levels predicted resistance to CDK4/6 inhibitors, they enhanced sensitivity to the PI3K/mTOR inhibitor Apitolisib. This therapeutic vulnerability was validated by SPR, which confirmed high-affinity binding between GUK1 and Apitolisib, and by cell viability assays where *GUK1* depletion induced drug resistance. **Conclusion:** GUK1 serves as a robust prognostic biomarker regulated by the TP53-HSF1 axis. Its distinct spatial patterns, immune-suppressive associations, and experimentally validated role in modulating PI3K/mTOR inhibitor sensitivity position GUK1 as a promising target for precision oncology in invasive BC.

## 1 Introduction

Breast cancer (BC) is the most frequently diagnosed cancer among women worldwide and the second leading cause of cancer-related death in the United States, with over 310,000 new cases and approximately 42,000 deaths projected in 2024, underscoring its significant public health burden [1,2]. Based on molecular classification, BC can be divided into subtypes including hormone receptor-positive, Human Epidermal Growth Factor Receptor 2 (HER2)-positive, and triple-negative breast cancer (TNBC), each associated with different treatment responses and outcomes. Current classification systems rely heavily on histopathological features to guide prognosis and therapy—particularly those involving the P53 and PI3K/AKT signaling pathways—and the tumor microenvironment in disease progression [3,4,5,6]. Furthermore, deciphering these underlying mechanisms, including the role of non-coding RNAs and novel therapeutic targets, remains essential for identifying new opportunities to suppress tumor growth [7,8,9,10]. Importantly, early detection through effective screening remains essential, as early-stage, non-metastatic disease is associated with significantly improved survival outcomes [11,12,13,14].

Despite advances in understanding BC subtypes, the role of specific metabolic enzymes like Guanylate kinase 1 (GUK1) in breast cancer remains underexplored. GUK1, also recognized as GMPK, is a vital enzyme involved in the metabolism of GTP and dGTP within cells. It plays a crucial role in both the recycling and *de novo* synthesis of purine [15]. GUK1 is closely associated with nucleic acid metabolism, significantly contributing to cell survival and proliferation processes. Further literature review reveals notable metabolic distinctions between cancer cells and normal cells, with alterations in these metabolic mechanisms potentially leading to accelerated tumor cell growth [16,17]. One such distinction was the heightened synthesis of nucleotides, resulting in increased levels of GTP and dGTP, which function as crucial precursor molecules for RNA and DNA synthesis [18]. Consequently, based on the comprehensive analysis of literature regarding cancer cell nucleic acid metabolism and the role of GUK1, inhibiting GUK1 activity is considered as a potentially effective strategy for suppressing tumor growth. Moreover, current research indicates that physiological functions of GUK1 extends to the activation of various antiviral and anticancer nucleoside analog prodrugs. These drugs included chemotherapeutic agents like 6-thioguanine and its analogue 6-mercaptopurine, as well as antiviral medications such as ganciclovir and acyclovir [19].

The relevance of GUK1 in breast cancer, particularly its overexpression and potential as a therapeutic target, is not well understood. In the current landscape of cancer research, our understanding of the role of GUK1 in human cancer cells remains limited. However, several studies have shed light on its potential significance in various cancer types. For instance, a study in non-small cell lung cancer (NSCLC) identifies GUK1 as a novel metabolic target within the ALK pathway. It is found that ALK interacts with GUK1, phosphorylating it to enhance intracellular nucleotide synthesis, specifically facilitating GDP/GTP synthesis. Molecular dynamics modeling techniques further reveal that phosphorylation induces conformational changes in the activation site of GUK1, making it more favorable for substrate interactions and preventing deactivation [20]. There have also been relatively few studies exploring GUK1 as a cancer biomarker. In glioma, a study integrating single-cell RNA sequencing data identifies strong correlations between GUK1 and the tumor feature vector encompassing all known glioma-related genes, suggesting its potential as a gene biomarker for glioma [21]. Additionally, in breast cancer metastasis, signal-based methods identified several genes, including GUK1, which are linked to cancer metastasis, cell invasion, and/or migration [22]. Although our understanding of GUK1 in human tumor cells is still evolving, these findings provide valuable practical insights into its potential role as a candidate gene biomarker in cancer.

The primary objective of this study was to identify functionally significant metabolic drivers in breast cancer through a systematic discovery-validation approach. We hypothesized that GUK1 acts as a central metabolic node associated with disease progression; accordingly, we investigated its clinical relevance and spatial localization, its regulation via the TP53-HSF1 axis, and its viability as a therapeutic target in PI3K-AKT-mTOR-driven tumors.

## 2 Materials & Methods

### 2.1 Candidate Gene Selection and Machine Learning-Based Feature Screening

To identify key metabolic drivers associated with tumor progression, we retrieved transcriptomic data and clinical information from the TCGA-BRCA dataset (1097 tumor samples and 114 normal samples) (https://portal.gdc.cancer.gov/). To ensure data consistency, high-throughput sequencing data (HTSeq-Counts) were downloaded and converted into Transcripts Per Million (TPM) to provide normalized expression levels for downstream comparative analysis. For metabolic characterization, genes with low abundance—defined as having a count of zero in more than 20% of the samples—were excluded from the initial matrix. The data underwent log_2_ transformation (log_2_(*TPM* + 1)) to reduce skewness and stabilize variance. To identify key metabolic drivers, we utilized the limma and DESeq2 R packages to perform differential expression analysis between tumor and normal cohorts, applying a Benjamini-Hochberg false discovery rate (FDR) correction (*p* < 0.05) and a fold-change threshold (|log_2_FC| > 1). Batch effects across different sequencing plates were scrutinized using Principal Component Analysis (PCA) and corrected where necessary using the ComBat algorithm from the sva package to ensure that the observed metabolic variations were biologically significant rather than technical artifacts. We focused specifically on metabolic reprogramming by curating a candidate list of 29 genes governing purine nucleotide metabolism.

To screen for genes that effectively distinguish between early-stage (AJCC Stage I and II) and late-stage (AJCC Stage III and IV) disease, we employed the Least Absolute Shrinkage and Selection Operator (LASSO) regression algorithm using the glmnet package (v4.1-8) in R (v4.5.2). This method minimizes the risk of overfitting by applying a penalty term to the regression coefficients. To determine the optimal tuning parameter (λ), we performed 10-fold cross-validation using the cv.glmnet function. To ensure the stability of the selected features and mitigate the impact of random data splitting, the cross-validation process was repeated 100 times, and the results were averaged to obtain a robust distribution of binomial deviance. We selected the λ value that yielded the minimum binomial deviance (λmin) as our primary selection criterion. This choice was justified by our objective to maximize the predictive accuracy of the identified metabolic drivers in distinguishing between early-stage and late-stage disease. The coefficient profiles of the 29 candidate genes were plotted against log(λ), and genes with non-zero coefficients at the optimal λ (determined by 10-fold cross-validation to minimize binomial deviance) were identified as key predictive features. To ensure the statistical confidence and stability of these coefficients, the LASSO selection process was repeated across 100 iterations of the training set, with GUK1 consistently emerging with the strongest positive coefficient, thereby indicating a robust association with tumor progression. To validate the predictive performance of the selected metabolic signature beyond the primary TCGA-BRCA cohort, we utilized High-Definition spatial transcriptomics (Visium HD) as an independent validation platform. This spatial analysis confirmed that GUK1 expression significantly colocalizes with expanding cancer cell nests and intensifies in advanced pathological stages, providing biological and cross-platform validation of the model’s top predictive feature.

### 2.2 Spatial Transcriptomics

Spatial transcriptomics analysis was performed using the Visium HD Human Transcriptome platform (10x Genomics, Inc., Pleasanton, CA, USA; https://www.10xgenomics.com/platforms/visium) on a commercial breast cancer tissue microarray (TissueArray.com LLC, cat# BR20818, Derwood, MD, USA), which meets ethical standards. To ensure a representative analysis of disease progression, we specifically selected and analyzed a total of six high-quality tissue cores, comprising three independent cores from patients with Stage I disease and three independent cores from patients with Stage III disease. The inclusion criteria for the commercial tissue microarray mandated that all samples be formal-fix-paraffin-embedded (FFPE) with a documented pathological diagnosis of invasive breast carcinoma. Prior to library preparation, RNA quality was assessed using the RNeasy FFPE kit (Qiagen, cat# 73504, Hilden, Germany), with all 6 selected tissue cores (three Stage I and three Stage III) successfully passing the inclusion threshold of DV200 > 30% on the Bioanalyzer (Agilent Technologies, Santa Clara, CA, USA). FFPE tissue sections (5 μm) were prepared following the Visium CytAssist Spatial Gene Expression protocol. Serial sections were H&E-stained and imaged using an Olympus V200 scanner (Olympus Life Science, Tokyo, Japan) at 20× magnification. To ensure high-resolution spatial mapping, the whole-slide images were acquired at a resolution of 0.274 μm/pixel using a high-resolution sCMOS camera, with subsequent image management and analysis performed using the OlyVIA (v3.4.1) software platform. The Visium CytAssist instrument (10x Genomics, Inc., Pleasanton, CA, USA) was used to facilitate the spatial transfer of transcriptomic probes from standard glass slides to Visium HD slides. This process allowed for the precise alignment of the H&E-stained tissue morphology with the capture area of the spatial discovery slide. Library preparation proceeded according to the Visium CytAssist protocol (CG000495), including probe hybridization, ligation, release, extension, and pre-amplification steps. During this process, spatial barcoding enabled preservation of positional information for each transcript.

Libraries were sequenced on an Illumina NovaSeq X plus platform with 150-base paired-end reads, targeting 600M reads per sample. Data analysis was performed using Space Ranger (v3.0) software (10x Genomics, Inc., Pleasanton, CA, USA), with alignment to the GRCh38-2020-A human reference genome. To ensure data quality, we applied a filtering threshold to exclude spots with fewer than 500 detected genes or high mitochondrial gene content (>15%). Normalization was conducted using the SCTransform algorithm to stabilize variance and account for differences in sequencing depth across the tissue sections. Batch effect correction was not required as all Stage I and Stage III cores were processed within the same Visium HD run to minimize technical variation. Cluster definition was achieved through Graph-based clustering and Principal Component Analysis (PCA), using the top 30 dimensions to resolve distinct anatomical and pathological regions. For visualization, gene expression heat maps and spatial feature plots were generated using Loupe Cell Browser (v7.0, 10x Genomics), with thresholds set at a minimum log_2_ fold-change of 1.0 and an adjusted *p*-value < 0.05 to identify spatially variable genes.

### 2.3 TNMplot

Differential gene expression between normal and tumor tissues was analyzed using TNMplot (https://tnmplot.com/analysis/) with the RNA-seq dataset for breast cancer [23]. In our research, we used the Gene vs. Gene Correlation panel of TNMplot to examine the association between *GUK1* expression and *HSF1*, which were identified from cBioPortal-based mutation group comparisons. Correlation analysis was performed using Pearson correlation on the RNA-seq dataset for breast cancer tissue. The TNMplot platform computes statistical significance using Pearson correlation coefficients, with multiple testing correction applied via the Benjamini–Hochberg FDR method.

### 2.4 Human Protein Atlas

The Human Protein Atlas (HPA) (https://www.proteinatlas.org/) is a comprehensive resource integrating immunohistochemistry (IHC), transcriptomics, and single-cell data to map protein expression across normal and cancerous human tissues [24]. We utilized the Pathology panel to validate the differential protein expression of GUK1 and HSF1 in normal versus tumor tissues based on IHC-stained tissue microarrays. The antibodies used for IHC staining were HPA048587 for GUK1 and HPA008888 for HSF1.

### 2.5 Single-Cell Transcriptomic Data Mining via TISCH2

To dissect the cellular heterogeneity of GUK1 expression within the breast cancer tumor microenvironment, we utilized the Tumor Immune Single-cell Hub 2 (TISCH2) database, a comprehensive web-based platform for single-cell RNA-sequencing (scRNA-seq) data mining. We specifically focused on two independent datasets, BRCA_GSE148673 and BRCA_EMTAB8107, to ensure the reproducibility of our findings. Raw scRNA-seq data from these cohorts were visualized using Uniform Manifold Approximation and Projection (UMAP) to identify major cell lineages, including malignant epithelial cells, fibroblasts, endothelial cells, and diverse immune populations such as B-cells, T-cells, and monocyte/macrophages. GUK1 transcript distribution was projected onto these UMAP clusters to identify cell-type-specific enrichment, specifically highlighting high expression within the monocyte/macrophage and CD8^+^ T-cell populations. For functional characterization, we employed the Gene Set Variation Analysis (GSVA) module in TISCH2 to calculate activity scores for the HALLMARK_IL6_JAK_STAT3_SIGNALING pathway across all identified clusters. In the EMTAB8107 cohort, GUK1 expression was mapped with high granularity to the “CD8Tex” sub-cluster to evaluate its association with exhausted CD8^+^ T-cells. This multi-cohort single-cell approach provided high-resolution evidence of the role of GUK1 in metabolic reprogramming and immune dysfunction within the breast cancer microenvironment.

### 2.6 UALCAN

UALCAN (https://ualcan.path.uab.edu/index.html) is an interactive web resource built on TCGA database, which enables comprehensive analysis of gene expression, promoter methylation, and clinical data across 33 cancer types [25,26]. In this study, we analyzed the expression levels of GUK1 and HSF1 in both tumor and normal breast tissues, as well as the promoter methylation levels of HSF1 in BC patients with and without TP53 mutations, using the TCGA module of the UALCAN database. The TCGA-BRCA dataset includes 1097 primary tumor samples and 114 adjacent normal breast tissue samples. Differential expression between tumor and normal tissues was analyzed using the UALCAN platform, which applies Student’s *t*-test to compare gene expression levels.

### 2.7 TISIDB

The TISIDB database is a comprehensive web portal that integrates multiple data types to investigate the interactions between tumors and the immune system, based on high-throughput data analysis and literature mining [27]. The lymphocyte and immunomodulator modules of the TISIDB database were used to evaluate the correlations between *GUK1* expression and immune cell infiltration as well as immune-related receptor genes in BRCA. The immune cell types analyzed included activated CD8^+^ T cells, activated CD4^+^ T cells, regulatory T cells, natural killer cells, B cells, macrophages, neutrophils, and dendritic cells. The immune-related receptor genes examined were *CTLA4*, *PDCD1*, *TGFB1*, *LAG3*, *CD28*, *CD80*, *ENTPD1*, and *NT5E*. Correlations were calculated using Spearman’s rank correlation.

### 2.8 Gene Set Cancer Analysis (GSCA)

Gene Set Cancer Analysis (GSCA) (https://guolab.wchscu.cn/GSCA/#/) represents an enhanced version of GSCALite, serving as a comprehensive database for exploring gene set cancer analysis related to mRNA expression, mutation, immune infiltration, and drug resistance [28]. In this study, the Immune Cell Abundance module of the GSCA platform was used to evaluate the correlation between GUK1 expression and exhausted T-cell infiltration in BRCA. In GSCA, exhausted T cells are estimated based on predefined gene signatures of T-cell exhaustion. Correlations were calculated using Spearman’s rank correlation and the analysis was restricted to the TCGA-BRCA cohort. We further generated trend plots from the TCGA dataset to examine stage-wise correlations between GUK1 and the expression of HSF1, Cyclin Dependent Kinase 4 (CDK4), Cyclin Dependent Kinase 6 (CDK6), and Cyclin D3 (CCND3) across tumor stages. Stage-specific correlations were calculated within each tumor stage subgroup based on TCGA clinical annotations. In addition, we investigated the associations between GUK1 and HSF1 expression and key cellular pathways, including DNA damage response and cell cycle activity. In GSCA, pathway activity scores are derived from predefined gene sets, and correlations between gene expression and pathway scores were used to assess potential functional associations.

### 2.9 Tumor Immune Estimation Resource (TIMER)

TIMER is a public web resource for comprehensive evaluation of the clinical impact of different immune cells in diverse cancer types [29]. TIMER2.0 (http://timer.cistrome.org/) is a web-based platform for systematic analysis of tumor-infiltrating immune cells and gene expression profiles across TCGA cancer types [30]. In this study, we used the Gene_DE module of TIMER2.0 to analyze the differential expression of *GUK1* across multiple TCGA cancer types. This analysis represents a pan-cancer comparison of tumor and normal tissues derived from TCGA datasets. Differential expression was evaluated using the Wilcoxon test implemented in TIMER2.0, and statistical significance was determined based on the default thresholds provided by the platform.

### 2.10 GEPIA2

The updated version of the Gene Expression Profiling Interactive Analysis (GEPIA2) database (http://gepia2.cancer-pku.cn/#index) has been developed to offer rapid and customizable features using TCGA and GTEx datasets [31]. We used the Survival Analysis module of GEPIA2 to evaluate disease-free survival (DFS) in relation to GUK1 expression across multiple cancer types in a pan-cancer analysis. Patients were stratified into high- and low-expression groups using the median GUK1 expression level as the cutoff. DFS was defined according to the TCGA clinical annotations implemented in GEPIA2. Survival differences were evaluated using Kaplan–Meier analysis with the log-rank test. Because the analysis focused on a predefined gene rather than genome-wide screening, multiple testing correction was not applied.

### 2.11 Kaplan-Meier Plotter

The Kaplan-Meier Plotter (https://kmplot.com/analysis/) is a web-based tool that evaluates the association between gene expression and survival outcomes across various cancer types, integrating data from GEO, EGA, and TCGA databases [32]. In this study, the mRNA gene chip panel of the Kaplan–Meier Plotter was used to analyze the association between GUK1 expression and survival outcomes in breast cancer patients. Patients were stratified into high- and low-expression groups according to the median expression level of GUK1. Overall survival (OS) was defined as the time from diagnosis to death from any cause, and relapse-free survival (RFS) was defined as the time from diagnosis to disease recurrence or progression. Survival differences were evaluated using Kaplan–Meier analysis with the log-rank test implemented in the platform. No additional clinical covariates were adjusted for in this analysis. Because the Kaplan–Meier Plotter integrates datasets from GEO, EGA, and TCGA, partial overlap with TCGA-derived datasets used elsewhere in this study may exist.

### 2.12 The Cancer Dependency Map

The Cancer Dependency Map (https://depmap.org/portal/) is a comprehensive resource that integrates functional genomics data, including RNA interference (RNAi) and CRISPR-Cas9 knockout screens, to identify genetic dependencies and therapeutic vulnerabilities in cancer cells [33,34]. In our research, we specifically filtered the DepMap database (Public 24Q2 and 24Q4 releases) to analyze data exclusively from human breast cancer cell lines. To evaluate the association between *HSF1* perturbation and *GUK1* expression, gene essentiality and perturbation effects were derived from genome-wide CRISPR-Cas9 knockout screens, with dependency quantified using the Chronos gene effect scoring algorithm. Corresponding baseline gene expression levels for *GUK1* and *HSF1* were obtained from the Cancer Cell Line Encyclopedia (CCLE) RNA-sequencing datasets within DepMap and quantified as log2(Transcripts Per Million (TPM) + 1). The relationship between *HSF1* CRISPR gene effect scores and *GUK1* transcript levels was statistically assessed using Pearson correlation analysis with two-tailed *p*-values. Additionally, the associations between *GUK1* and *HSF1* expression and PI3K–AKT–mTOR pathway activity were evaluated using predefined pathway activity scores. Drug sensitivity data for CDK4/6 inhibitors (e.g., Palbociclib) were obtained from the DepMap PRISM drug repurposing dataset (Sanger GDSC1), where cellular sensitivity was quantified using Area Under the Dose-Response Curve (AUC) values. Pearson and Spearman correlation analyses were used to statistically evaluate the associations between *GUK1* expression, pathway activities, and drug sensitivity metrics. 

### 2.13 Q-omics

The Q-omics platform is a bioinformatics tool that enables data mining and visualization of multi-omics datasets in oncology research. It integrates standardized datasets from The Cancer Genome Atlas (TCGA) and the Genomics of Drug Sensitivity in Cancer (GDSC) database [35]. The platform’s algorithmic framework standardizes cross-database queries to link gene expression profiles with pharmacological response metrics. In this study, we used Q-omics to evaluate the correlation between *GUK1* expression and drug sensitivity to Uprosertib and Apitolisib, two small-molecule inhibitors targeting the PI3K–AKT–mTOR pathway. The analysis parameters were configured as follows: the queried cancer lineage was strictly filtered to “Breast”; baseline gene expression was quantified as log_2_(Transcripts Per Million (TPM) + 1); and drug sensitivity was evaluated utilizing the negative logarithm of the half-maximal inhibitory concentration (−log(IC50)); sample split was set at median.

### 2.14 Cell Culture and siRNA Transfection

The human triple-negative breast cancer cell line MDA-MB-231 was obtained from the American Type Culture Collection (HTB-26, ATCC, Manassas, VA, USA). Prior to use in experiments, the cell line was authenticated via Short Tandem Repeat (STR) DNA profiling to verify its genetic identity. Additionally, the cells were routinely screened for mycoplasma contamination using a PCR-based detection assay and consistently tested negative. The cells were cultured in Dulbecco’s Modified Eagle’s Medium (DMEM; 11965-092, Gibco, Thermo Fisher Scientific, Waltham, MA, USA) supplemented with 10% fetal bovine serum (FBS; 16000-044, Gibco) and 1% penicillin–streptomycin (15140-122, Gibco) in a humidified incubator at 37°C with 5% CO_2_. 20 nM of small interfering RNAs (siRNAs) targeting GUK1 (ID: s6537; sense: 5′-GGCUUCAGCGUGUCCCAUAtt-3′; anti-sense: 5′-UAUGGGACACGCUGAAGCCaa-3′) and HSF1 (ID: s6951; sense: 5′-GCUUCCACGUGUUCGACCAtt-3′; anti-sense: 5′-UGGUCGAACACGUGGAAGCtg-3′), along with a negative control siRNA (si-ctrl; ID: 4390843; a proprietary non-targeting sequence with no known human homology), were synthesized by Thermo Fisher Scientific. Cells were seeded at a density of 2.5 × 10^4^ in 24-well plates. Upon reaching 60–70% confluence, the cells were transfected using Lipofectamine 3000 (L3000008, Invitrogen, Thermo Fisher Scientific) according to the manufacturer’s instructions. Briefly, for each well, 10 pmol of siRNA and 1.5 μL of Lipofectamine 3000 reagent were separately diluted in 25 μL of Opti-MEM Reduced Serum Medium. The diluted siRNA and Lipofectamine 3000 were then mixed to form transfection complexes (50 μL total volume) and incubated for 15 min at room temperature. The 50 μL complex was added dropwise to the cells cultured in 450 μL of antibiotic-free DMEM, yielding a final siRNA concentration of 20 nM in a total reaction volume of 500 μL per well. Knockdown efficiency was confirmed by RT-qPCR 48 h post-transfection, with 18S rRNA as an endogenous control.

### 2.15 Wound Healing Assay

The wound healing assay was performed following a previously described protocol with minor modifications [36]. Following the siRNA transfection procedure described in Section 2.14, cells were incubated for 72 h. Upon verifying that the cells had formed a nearly 95–100% confluent monolayer to ensure uniform migration conditions, a linear wound was created in each well using a 10 μL pipette tip. Detached cells and debris were removed by washing twice with 1× PBS. To minimize the confounding effects of cell proliferation during the migration assay, the cells were subsequently cultured in reduced-serum medium (DMEM supplemented with 1% FBS). Wound closure was monitored at 0 and 24 h using a Lionheart FX microscope (Agilent Technologies, CA, USA). The wound area was quantified using ImageJ software (v1.53; National Institutes of Health, Bethesda, MD, USA).

### 2.16 Bulk RNA-Seq and Pathway Analysis

Total RNA extracted from cell lysate was quantified using a NanoDrop spectrophotometer (Thermo Fisher Scientific, Waltham, USA) and integrity was assessed with an Agilent 2100 Bioanalyzer and RNA 6000 Nano Kit (Cat. No. 5067-1511; Agilent Technologies, Santa Clara, USA). To ensure optimal reproducibility and adequate library preparation, only RNA samples with an RNA Integrity Number (RIN) ≥ 7.0 were utilized for downstream applications. One microgram RNA per sample was treated with TURBO DNA-free Kit (AM1907, Invitrogen, Carlsbad, USA), followed by poly(A) mRNA enrichment using the QIAseq Stranded mRNA Select Kit (180746, QIAGEN, Hilden, Germany). RNA-seq libraries were prepared with the QIAseq UPXome RNA Library Kit (Cat. No. 334702, QIAGEN, Hilden, Germany), quality-controlled by Qubit dsDNA HS Assay (Cat. No. Q32851, Thermo Fisher Scientific) and Bioanalyzer DNA 1000 Kit (Cat. No. 5067-1504, Agilent Technologies, Santa Clara, CA, USA), and sequenced on an Illumina NovaSeq X Plus (2 × 151 bp paired-end; Illumina, San Diego, USA). Raw reads were trimmed (QV ≥ 20, no ambiguous bases, TruSeq adapter removal) and aligned to the human reference genome (hg38) using the built-in RNA-Seq analysis tool in CLC Genomics Workbench v10 (QIAGEN, Hilden, Germany) with default mapping parameters (mismatch cost: 2, insertion cost: 3, deletion cost: 3, length fraction: 0.8, similarity fraction: 0.8, maximum number of hits per read: 10). Multi-mapping reads were assigned probabilistically using the built-in EM algorithm. Expression values were calculated as FPKM (fragments per kilobase of exon per million mapped fragments). To perform gene set enrichment analysis (GSEA), the entire list of expressed genes was ranked based on their log_2_ fold-change values. The ranked continuous gene list was then subjected to GSEA using curated Gene Ontology (GO) Biological Process and KEGG gene sets from the Molecular Signatures Database (MSigDB). Pathways with a False Discovery Rate (FDR) adjusted *p*-value < 0.05 were considered significantly enriched.

### 2.17 Cell Viability and Drug Sensitivity Assay

Cell viability was assessed using the Cell Counting Kit-8 (CCK-8) assay (Cat. No. S2287, Selleck Chemicals, Houston, TX, USA). Transfected cells (si-ctrl and si-GUK1) were seeded into 96-well plates (3 × 10^3^ cells/well) and treated with increasing concentrations of Apitolisib (0, 0.078, 0.156, 0.312, 0.625, 1.25, 2.5, 5 and 10 μM; S2786, Selleck Chemicals, Houston, TX, USA) for 48 h. CCK-8 reagent was added to each well, and absorbance was measured at 450 nm using a SpectraMax iD3 microplate reader (Molecular Devices, San Jose, CA, USA). To ensure accurate quantification, the background absorbance from blank wells (containing culture medium and CCK-8 reagent without cells) was subtracted from the optical density (OD) readings of all experimental wells. Relative cell viability was then calculated as a percentage of the background-corrected absorbance of the vehicle-treated control cells. The half-maximal inhibitory concentration (*IC*_50_) was calculated using non-linear regression analysis in GraphPad Prism, specifically applying a four-parameter logistic (4PL) curve-fitting model (log(inhibitor) vs. normalized response with variable slope).

### 2.18 Protein Extraction and Western Blotting

Cells were washed with ice-cold PBS and lysed in RIPA lysis buffer (Cat. No. 89901, Thermo Fisher Scientific, Waltham, MA, USA) supplemented with protease and phosphatase inhibitor cocktails (Cat. No. 78440, Thermo Fisher Scientific, Waltham, MA, USA) to prevent protein degradation and preserve phosphorylation states. The cell lysates were centrifuged at 12,000× *g* for 15 min at 4°C, and the supernatants were collected. Total protein concentrations were determined using a BCA protein assay kit (Cat. No. 23227, Thermo Fisher Scientific, Waltham, MA, USA).

Equal amounts of protein samples (50 μg) were separated by SDS-PAGE on 7% polyacrylamide gels and electro-transferred onto polyvinylidene fluoride (PVDF) membranes (Cat. No. BSP0161, Pall Corporation, Port Washington, NY, USA). After blocking with 5% bovine serum albumin (BSA) in Tris-buffered saline containing 0.1% Tween 20 (TBST) for 1 h at room temperature, the membranes were incubated overnight at 4°C with the following primary antibodies: anti-p-AKT (Ser473) (GTX128414, 1:1000, GeneTex, Irvine, CA, USA), anti-AKT (GTX640258, 1:1000, GeneTex, Irvine, CA, USA), anti-p-mTOR (Ser2448) (GTX132803, 1:1000, GeneTex, Irvine, CA, USA), anti-mTOR (GTX638220, 1:1000, GeneTex, Irvine, CA, USA), and anti-β-actin (GTX638580, 1:5000, GeneTex, Irvine, CA, USA).

Following three washes with TBST, the membranes were incubated with horseradish peroxidase (HRP)-conjugated secondary antibodies, including Peroxidase AffiniPure^®^ Goat Anti-Rabbit IgG (H + L) (1:5000, Jackson ImmunoResearch, West Grove, PA, USA) and Peroxidase AffiniPure^®^ Goat Anti-Mouse IgG (H + L) (1:5000, Jackson ImmunoResearch, West Grove, PA, USA), for 1 h at room temperature. Protein bands were detected using LumiFlash™ Infinity Chemiluminescent Substrate, HRP System (LF16-500, Visual Protein, Taipei, Taiwan) and visualized using a chemiluminescence imaging system (ChemiDoc MP, Bio-Rad Laboratories, Hercules, CA, USA). The densitometry of the specific protein bands was quantified using ImageJ software (v1.53; National Institutes of Health, Bethesda, MD, USA). 

### 2.19 Molecular Docking Analysis

To investigate the potential direct interaction between GUK1 and the PI3K/mTOR inhibitor Apitolisib, we performed an integrated molecular docking study. The three-dimensional protein structure of human GUK1 (UniProt ID: Q16774) was retrieved from the AlphaFold Protein Structure Database (https://alphafold.ebi.ac.uk/) to ensure a high-confidence structural model. The chemical structure of Apitolisib (GDC-0980) was obtained from the PubChem database (https://pubchem.ncbi.nlm.nih.gov/) and prepared for docking using OpenBabel (version 3.1.1; Open Babel Development Team; http://openbabel.org) to minimize energy and optimize geometry. Molecular docking simulations were conducted using PyRx virtual screening software (version 0.8; Sarkis Dallakyan, San Diego, CA, USA), utilizing the AutoDock Vina algorithm. The GUK1 protein was treated as a rigid receptor, while the Apitolisib ligand was allowed to have rotatable bonds. A grid box was defined to encompass the predicted active site of GUK1, centered to maximize the search space for potential binding pockets. To validate the reliability of the docking protocol, the known endogenous substrate of GUK1, guanosine monophosphate (GMP), was initially docked into the structural model. This validation successfully targeted the core nucleotide monophosphate (NMP) binding domain with a highly favorable binding affinity (−8.726 kcal/mol) (Supplementary Fig. S1), confirming the accuracy of the grid box and computational parameters. Subsequently, the docking results for Apitolisib were evaluated based on binding affinity (kcal/mol), with the most negative values indicating the most thermodynamically stable conformations. Post-docking analysis and visualization of the protein-ligand interactions were performed using LigPlot+ and PyMOL. Key interactions, including hydrogen bonds and hydrophobic contacts between the ligand and specific amino acid residues, were identified to elucidate the structural basis of the binding mechanism.

### 2.20 Surface Plasmon Resonance (SPR) Analysis

The binding affinity between the GUK1 protein and Apitolisib was measured using a Biacore system (Cytiva, Marlborough, MA, USA) at a constant temperature of 25°C. Recombinant human GUK1 protein (50 μg/mL in sodium acetate buffer, pH 4.5) was covalently immobilized on a CM5 sensor chip (Cytiva) via a standard amine coupling procedure. Briefly, flow cell 2 of the sensor chip was activated using a mixture of 1-ethyl-3-(3-dimethylaminopropyl)carbodiimide (EDC) and N-hydroxysuccinimide (NHS). The GUK1 protein was then injected at a flow rate of 10 μL/min, followed by a blocking step using ethanolamine to deactivate the remaining reactive esters. Apitolisib was diluted in running buffer (1× PBS, pH 7.4, containing 5% DMSO) to various concentrations (0.3125, 0.625, 1.25, 2.5, 5 and 10 μM) and injected over the chip surface at a flow rate of 30 μL/min. The association and dissociation phases were monitored in real-time for 60 s and 90 s, respectively. After each cycle, the sensor chip surface was regenerated using 10 mM glycine-HCl (pH 2.0) for 5 min. The association and dissociation phases were monitored in real-time. Data were analyzed using Biacore Insight Evaluation Software, and the equilibrium dissociation constant (*K_D_*) was calculated using the 1:1 Langmuir binding model.

### 2.21 Statistical Analysis

Statistical analyses were performed using R software (v4.0.3). Differences between two groups were assessed using the Wilcoxon rank-sum test or Student’s *t*-test. For correlation analyses involving genome-wide or high-dimensional datasets (e.g., immune cell abundance estimates from ImmuCellAI and GSCA pathway activities), *p*-values were corrected for multiple hypothesis testing using the Benjamini-Hochberg False Discovery Rate (FDR) method. A *p*-value or FDR < 0.05 was considered statistically significant. Pearson correlation coefficients (r) were used to evaluate the strength of linear associations between gene expression levels.

## 3 Results

### 3.1 Identification of GUK1 as a Key Metabolic Driver of Tumor Progression via Machine Learning and Spatial Validation

Metabolic reprogramming is a recognized hallmark of cancer, facilitating the unrestricted cell proliferation required for tumor progression. Among these metabolic alterations, the dysregulation of nucleotide metabolism—specifically purine synthesis—is critical for providing the necessary substrates for DNA replication and RNA synthesis in rapidly dividing tumor cells. Consequently, we hypothesized that key enzymes driving this metabolic shift may serve as robust biomarkers for disease advancement. 

**Figure 1:**
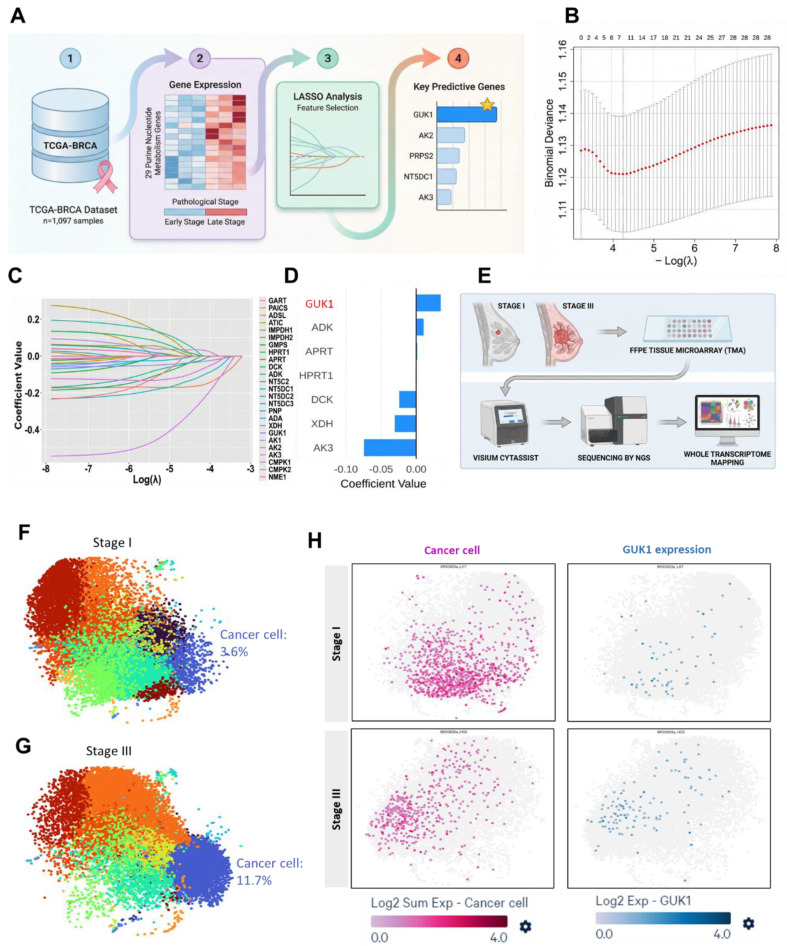
**Identification of Guanylate kinase 1 (*GUK1*) as a key prognostic biomarker in breast cancer through integrated multi-omics and spatial transcriptomics.** (**A**) Schematic workflow of the study. The TCGA-BRCA dataset (*n* = 1097) was analyzed to screen 29 purine nucleotide metabolism-related genes. Least Absolute Shrinkage and Selection Operator (LASSO) regression was employed to identify features distinguishing early-stage from late-stage disease, highlighting *GUK1* as a top candidate. The yellow star indicates the key predictive gene identified from the LASSO analysis. (**B**) Selection of the optimal tuning parameter (λ) for the LASSO model using 10-fold cross-validation. The dashed vertical lines represent the optimal λ values based on binomial deviance. (**C**) LASSO coefficient profiles of the 29 candidate genes against log(λ). (**D**) Bar plot showing the coefficients of the selected predictive genes. *GUK1* exhibits the strongest positive coefficient, indicating a positive association with tumor progression, while genes such as *AK3* show negative associations. (**E**) Schematic overview of the spatial transcriptomics validation workflow using Formalin-Fixed, Paraffin-Embedded (FFPE) Tissue Microarrays (TMA) and the 10x Genomics Visium CytAssist platform to compare Stage I and Stage III breast cancer tissues. (**F**,**G**) Spatial clustering maps of Stage I (**F**) and Stage III (**G**) tissues. The identified cancer cell cluster (highlighted in dark blue) expands from 3.6% in Stage I to 11.7% in Stage III. (**H**) Spatial feature plots visualizing the distribution of the cancer cell signature (magenta) and *GUK1* expression (blue) in Stage I and Stage III tissues. *GUK1* expression is markedly elevated in Stage III and shows spatial colocalization with cancer cell-rich regions.

To test this hypothesis, we adopted a targeted candidate gene approach rather than an unbiased transcriptome-wide search. We focused on 29 genes specifically governing purine nucleotide metabolism and analyzed their expression profiles within the TCGA-BRCA cohort (*n* = 1097) (Fig. 1A). To identify specific genes capable of distinguishing between early-stage and late-stage disease, we employed the Least Absolute Shrinkage and Selection Operator (LASSO) regression algorithm for feature selection. The LASSO analysis, optimized by binomial deviance minimization (Fig. 1B) and coefficient profiling (Fig. 1C), successfully filtered the candidate list. Among the identified predictors, Guanylate Kinase 1 (*GUK1*) emerged as the most significant feature associated with advanced pathological stages, exhibiting the strongest predictive weight among the selected metabolic enzymes (Fig. 1D). 

To validate these *in silico* findings in a clinical context and resolve spatial heterogeneity, we utilized High-Definition spatial transcriptomics (Visium HD) on breast cancer patient tissues (Fig. 1E). We specifically compared the spatial expression patterns of *GUK1* between early (Stage I) and advanced (Stage III) tumors. The single-cell resolution clustering revealed that the proportion of cancer cells expanded notably from 3.6% in Stage I (Fig. 1F) to 11.7% in Stage III (Fig. 1G). Crucially, *GUK1* expression was not randomly distributed; it strongly colocalized with these expanding cancer cell nests (Fig. 1H). The signal intensity of *GUK1* was markedly elevated in Stage III compared to Stage I, confirming that *GUK1* upregulation is an intrinsic feature associated with the expansion of the tumor compartment during disease progression.

### 3.2 GUK1 Upregulation as a Candidate Biomarker with Diagnostic and Prognostic Significance in BC

**Figure 2:**
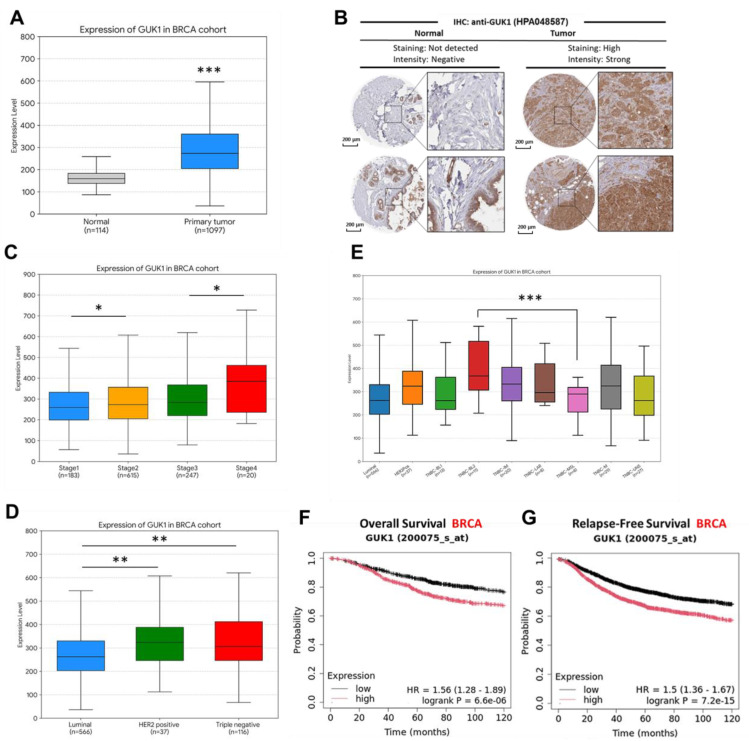
**Elevated *GUK1* expression correlates with tumor progression and poor prognosis in breast cancer.** (**A**) Box plot comparing GUK1 mRNA expression in normal breast tissues (*n* = 114) versus primary tumor tissues (*n* = 1097) in the BRCA cohort. (**B**) Immunohistochemistry (IHC) analysis (antibody HPA048587) showing representative images of GUK1 protein expression. Normal tissues show negative staining intensity, while tumor tissues display strong cytoplasmic staining. Scale bar: 200 μm. (**C**) Correlation of GUK1 expression with clinical stages (Stage 1–4), showing increased expression in advanced stages. (**D**) *GUK1* expression levels across major molecular subtypes: Luminal, HER2-positive, and Triple-negative (TNBC). (**E**) Detailed analysis of *GUK1* expression across distinct TNBC sub-clusters and expanded classifications. (**F**,**G**) Kaplan-Meier survival curves for Overall Survival (OS) (**F**) and Relapse-Free Survival (RFS) (**G**). Patients were stratified into low (black) and high (red) expression groups; Hazard Ratios (HR) and log-rank *p*-values are indicated. (*p < 0.05, **p < 0.01, ***p < 0.001).

To evaluate the clinical relevance of *GUK1* in breast cancer (BC), we analyzed its expression patterns and prognostic value using large-scale patient cohorts and tissue validation. Analysis of the BRCA cohort revealed that *GUK1* mRNA expression is significantly elevated in primary tumor tissues (*n* = 1097) compared to normal breast tissues (*n* = 114, *p* < 0.001; Fig. 2A). This upregulation was further validated at the protein level via immunohistochemistry (IHC), where tumor samples exhibited strong cytoplasmic intensity compared to the undetected staining in normal tissues (Fig. 2B). We further observed a correlation between *GUK1* expression and disease progression, with levels showing a significant stepwise increase from Stage 1 through Stage 4 (Fig. 2C). Moreover, *GUK1* expression was found to be subtype-dependent, with significantly higher levels observed in aggressive HER2-positive and TNBC subtypes compared to the Luminal subtype (Fig. 2D). A more granular analysis of TNBC sub-clusters highlighted distinct heterogeneity, with specific molecular clusters displaying marked *GUK1* upregulation (Fig. 2E). Kaplan-Meier survival analysis demonstrated that high *GUK1* expression is a strong predictor of poor outcomes, significantly correlating with reduced Overall Survival (HR = 1.56, *p* = 6.6 × 10^−6^; Fig. 2F) and Relapse-Free Survival (HR = 1.5, *p* = 7.2 × 10^−15^; Fig. 2G).

### 3.3 Single-Cell Profiling Identifies High GUK1 Expression in Immunosuppressive Myeloid and Exhausted T-Cell Populations

**Figure 3:**
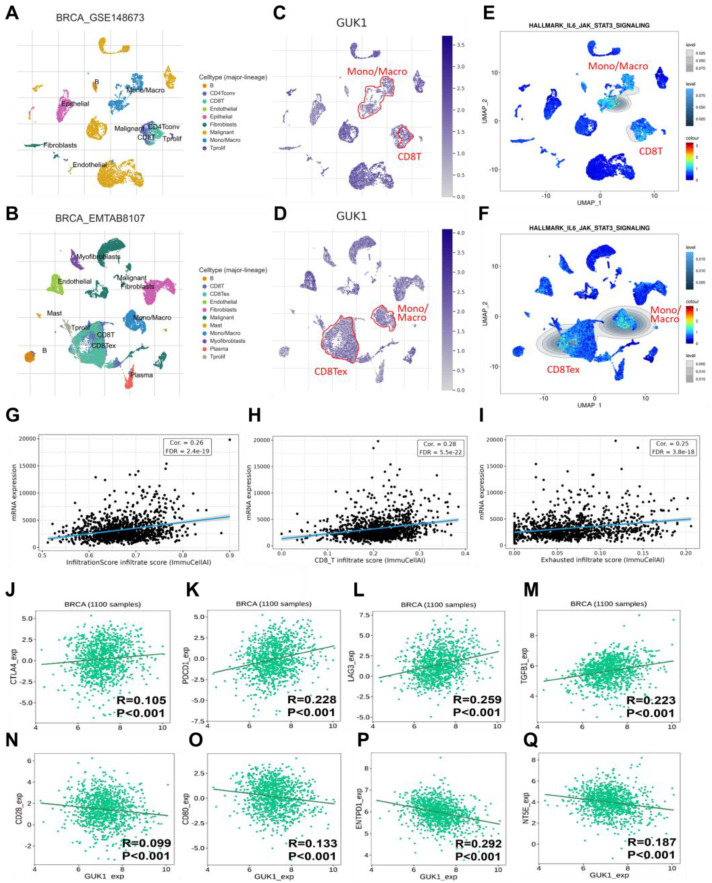
**Single-cell and bulk transcriptomic analysis reveals *GUK1* association with an immunosuppressive microenvironment and T-cell exhaustion.** (**A**–**F**) Single-cell analysis of breast cancer datasets GSE148673 (**A**,**C**,**E**) and EMTAB8107 (**B**,**D**,**F**). (**A**,**B**) UMAP plots visualizing major cell type clusters. (**C**,**D**) Distribution of *GUK1* expression across cell clusters. Red outlines indicate high *GUK1* expression in Monocyte/Macrophage (Mono/Macro) and CD8^+^ T-cell (CD8T/CD8Tex) populations. (**E**,**F**) Activity scores of the HALLMARK_IL6_JAK_STAT3_SIGNALING pathway projected onto the UMAP space, showing overlap with *GUK1*-high clusters. (**G**–**I**) Scatter plots showing the correlation between *GUK1* mRNA expression and immune signatures derived from ImmuCellAI in the TCGA cohort, including overall Infiltration Score (**G**), CD8^+^ T-cell infiltration (**H**), and Exhausted T-cell score (**I**). FDR values indicate statistical significance adjusted for multiple testing. (**J**–**Q**) Correlation analysis between *GUK1* expression and key immune checkpoint or immunosuppressive markers in TCGA-BRCA samples (*n* = 1100). Scatter plots display Pearson correlations for (**J**) Cytotoxic T-Lymphocyte Associated Protein 4 (*CTLA4*), (**K**) Programmed Cell Death 1 (*PDCD1*), (**L**) Transforming Growth Factor Beta 1 (*TGFB1*), (**M**) Lymphocyte Activating 3 (*LAG3*), (**N**) Cluster of differentiation 28 (*CD28*), (**O**) *CD80*, (**P**) Ectonucleoside Triphosphate Diphosphohydrolase 1 (*ENTPD1*), and (**Q**) 5′-Nucleotidase Ecto (*NT5E*). All *p*-values and correlation coefficients (**R**) are indicated in the respective panels.

Given the critical role of the microenvironment in tumor progression, metastasis, and immune evasion, we dissected the cellular heterogeneity of *GUK1* expression within the tumor microenvironment (TME), we analyzed single-cell RNA sequencing (scRNA-seq) data from two independent breast cancer cohorts (GSE148673 and EMTAB8107). Uniform Manifold Approximation and Projection (UMAP) clustering identified major stromal and immune lineages, including T-cells, B-cells, fibroblasts, and myeloid cells (Fig. 3A,B). While *GUK1* expression was detected in malignant cells, we observed a notable enrichment of *GUK1* transcripts within the monocyte/macrophage and CD8^+^ T-cell clusters (Fig. 3C,D).

Functional enrichment analysis on these clusters revealed that the *GUK1*-high immune populations displayed significantly elevated activity of the IL6-JAK-STAT3 signaling pathway (Fig. 3E,F), a cascade well-established in driving immunosuppression and tumor progression. In the EMTAB8107 cohort, *GUK1* expression was specifically prominent in the “CD8Tex” cluster, corresponding to exhausted CD8^+^ T-cells, suggesting a potential role for *GUK1* in metabolic reprogramming associated with T-cell dysfunction.

We validated these single-cell findings using bulk RNA-seq data from the TCGA-BRCA cohort (n = 1100). *GUK1* mRNA levels showed a positive correlation with overall immune infiltration scores (Cor = 0.26, FDR = 2.4 × 10^−19^) and CD8^+^ T-cell infiltration (Cor = 0.28, FDR = 5.5 × 10^−22^) (Fig. 3G,H). However, this infiltration appears to be functionally compromised, as *GUK1* expression was also significantly correlated with the “Exhausted” immune cell score (Fig. 3I).

Further correlation analysis confirmed a robust association between *GUK1* and key markers of immune exhaustion and suppression. We observed significant positive correlations between *GUK1* and inhibitory checkpoint receptors, including *CTLA4* (R = 0.105), *PDCD1* (PD-1; R = 0.228), and *LAG3* (R = 0.259) (Fig. 3J–L). Additionally, *GUK1* expression tracked with the immunosuppressive cytokine *TGFB1* (R = 0.223) (Fig. 3M) and immunoreactive molecule CD28 (Fig. 3N) and CD80 (Fig. 3O) and components of the adenosine signaling pathway, *ENTPD1* (CD39; R = 0.292) (Fig. 3P) and *NT5E* (CD73; R = 0.187) (Fig. 3Q). Collectively, these multi-omics data suggest that *GUK1* overexpression fosters an immunosuppressive microenvironment characterized by macrophage polarization and CD8^+^ T-cell exhaustion, potentially limiting the efficacy of anti-tumor immunity.

### 3.4 HSF1 Promoter Hypomethylation Correlates with HSF1 Overexpression and Positive Regulation of GUK1

**Figure 4:**
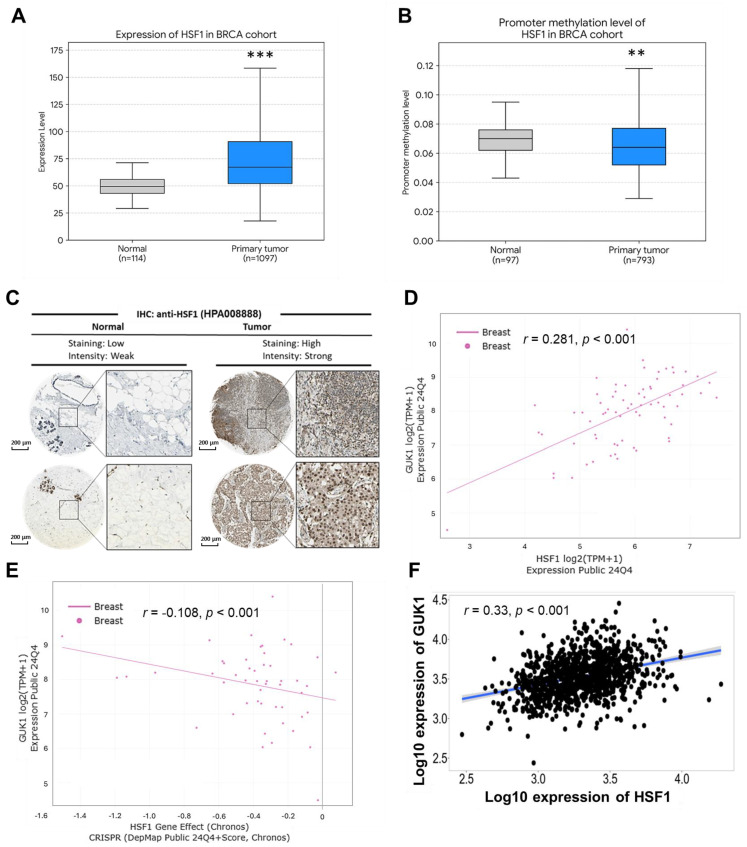
**Epigenetic upregulation of *HSF1* is associated with *GUK1* overexpression in breast cancer.** (**A**,**B**) Analysis of TCGA samples reveals (**A**) significantly elevated HSF1 mRNA expression in primary tumors (*n* = 1097) compared to normal tissue (n = 114), consistent with (**B**) significant promoter hypomethylation in the tumor group. (**C**) Representative IHC images from the Human Protein Atlas (antibody HPA008888) confirm stronger HSF1 protein staining in tumor tissues compared to normal controls. Scale bar: 200 μm. (**D**,**E**) Analysis of breast cancer cell lines using the DepMap portal (Public 24Q4) shows (**D**) a positive correlation between *HSF1* and *GUK1* transcript levels (*r* = 0.281, *p* < 0.001) and (**E**) a negative correlation between *HSF1* CRISPR gene effect scores and GUK1 expression (*r* = −0.108, *p* < 0.001), suggesting that cell lines with higher *GUK1* expression are more dependent on *HSF1*. (**F**) Scatter plot verifying the positive correlation between HSF1 and GUK1 expression in the clinical breast cancer cohort (*r* = 0.33, *p* < 0.001). Statistical significance: **p < 0.01, ***p < 0.001.

To elucidate the regulatory mechanisms driving *GUK1* overexpression, we investigated the role of Heat Shock Factor 1 (*HSF1*), a key transcription factor previously implicated in stress response and oncogenesis. Analysis of RNA sequencing data from the TCGA-BRCA cohort revealed a significant upregulation of *HSF1* mRNA expression in primary tumor tissues compared to normal breast tissues (Fig. 4A). This transcriptional increase was accompanied by a significant reduction in *HSF1* promoter methylation levels in the tumor group (Fig. 4B), suggesting that epigenetic hypomethylation contributes to *HSF1* overexpression in breast cancer. At the protein level, immunohistochemical validation using data from the Human Protein Atlas confirmed that *HSF1* exhibits strong nuclear staining intensity in tumor tissues, contrasting with the weak or undetectable staining observed in normal controls (Fig. 4C).

We further explored the functional relationship between *HSF1* and *GUK1* using the Cancer Dependency Map (DepMap). In breast cancer cell lines, we observed a statistically significant positive correlation between *HSF1* and *GUK1* transcript levels (*r* = 0.281, *p* < 0.001; Fig. 4D). Moreover, analysis of CRISPR-Cas9 knockout screens revealed a negative correlation between *GUK1* expression and *HSF1* gene effect scores (Chronos) (*r* = −0.108, *p* < 0.001; Fig. 4E). Since more negative Chronos scores indicate a stronger gene essentiality, this finding suggests that breast cancer cell lines with high *GUK1* expression possess an increased dependency on *HSF1* for survival. Finally, we validated the co-expression of these genes in clinical samples, confirming a robust positive correlation between *HSF1* and *GUK1* mRNA levels in the breast cancer cohort (*r* = 0.33, *p* < 0.001; Fig. 4F).

### 3.5 Upregulation of HSF1 and GUK1 in TP53 Mutation Status and Their Positive Correlation with DNA Damage Activity

**Figure 5:**
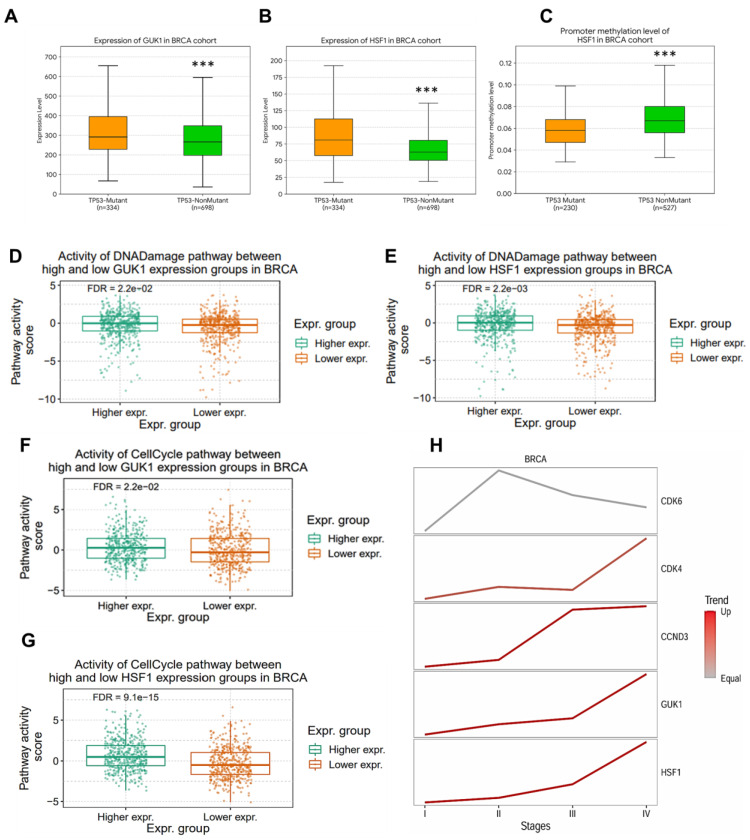
**Interplay of *HSF1* and *GUK1* with *TP53* mutation status and cell cycle regulation in breast cancer.** (**A**–**C**) Analysis of *GUK1* and *HSF1* relative to *TP53* mutation status. Box plots display the gene expression levels of (**A**) *GUK1* and (**B**) *HSF1*, as well as (**C**) *HSF1* promoter methylation levels in normal tissue (n = 334, 334, and 230, respectively) compared to *TP53*-mutant and *TP53*-non-mutant breast cancer samples (n = 698, 698, and 527, respectively). (**D**–**G**) Box plots comparing pathway activity scores between high and low gene expression groups. DNA Damage pathway activity is significantly associated with high expression of (**D**) *GUK1* and (**E**) *HSF1*. (**F**,**G**) Cell Cycle pathway activity is similarly elevated in patients with high expression of (**F**) *GUK1* and (**G**) *HSF1*. (**H**) Trend plot derived from TCGA data showing the expression trajectories of *GUK1*, *HSF1*, and key cell cycle regulators: Cyclin-Dependent Kinase 4, Cyclin-Dependent Kinase 6, and Cyclin D3 (*CDK4*, *CDK6*, and *CCND3*) across breast cancer tumor stages (I–IV). Statistical significance: ***p < 0.001 vs. normal or non-mutant groups. FDR, False Discovery Rate. All data derived from TCGA datasets.

Having established the *HSF1*-*GUK1* regulatory axis, we sought to identify the upstream drivers initiating this mechanism. Given that *TP53* mutations are frequent drivers of genomic instability and metabolic rewiring in breast cancer, we investigated their impact on this axis. Analysis of the TCGA-BRCA cohort stratified by *TP53* status revealed that patients harboring *TP53* mutations exhibited significantly higher expression levels of both *GUK1* (Fig. 5A) and *HSF1* (Fig. 5B) compared to *TP53*-wild-type tumors and normal tissues. Mechanistically, this upregulation in *TP53*-mutant tumors was accompanied by a significant decrease in *HSF1* promoter methylation (Fig. 5C). To explore the biological basis underlying this epigenetic alteration, we evaluated the expression of core DNA methyltransferases in relation to TP53 status. Analysis of the TCGA-BRCA cohort revealed that tumors harboring TP53 mutations exhibited significant dysregulation in the mRNA expression levels of DNMT1, DNMT3A, and DNMT3B compared to TP53 wild-type tumors (Wilcoxon rank-sum test, *p* < 0.001; Supplementary Fig. S2). This finding reinforces the hypothesis that loss of functional *TP53* may facilitate *HSF1* promoter hypomethylation, thereby releasing its transcriptional suppression and driving downstream *GUK1* overexpression.

To understand the functional implications of this axis, we evaluated its association with key oncogenic pathways. High expression of both *GUK1* and *HSF1* was significantly associated with elevated DNA Damage Response pathway activity (Fig. 5D,E), consistent with the requirement for upregulated nucleotide metabolism to maintain genomic integrity under replicative stress. Furthermore, both genes showed a significant positive correlation with Cell Cycle pathway activity scores (Fig. 5F,G).

This association with proliferation was further corroborated by stage-dependent expression trajectories. Trend analysis demonstrated that *GUK1* and *HSF1* expression levels progressively increase from Stage I to Stage IV, mirroring the upregulation of key cell cycle regulators such as *CDK4* and *CCND3* (Fig. 5H). In contrast, *CDK6* did not exhibit a similar sustained upregulation, suggesting that the *TP53*-*HSF1*-*GUK1* axis specifically co-regulates with a distinct subset of proliferation markers to drive aggressive disease progression.

### 3.6 The TP53-HSF1 Axis Regulates GUK1 to Drive Cell Migration and mTOR Signaling

**Figure 6:**
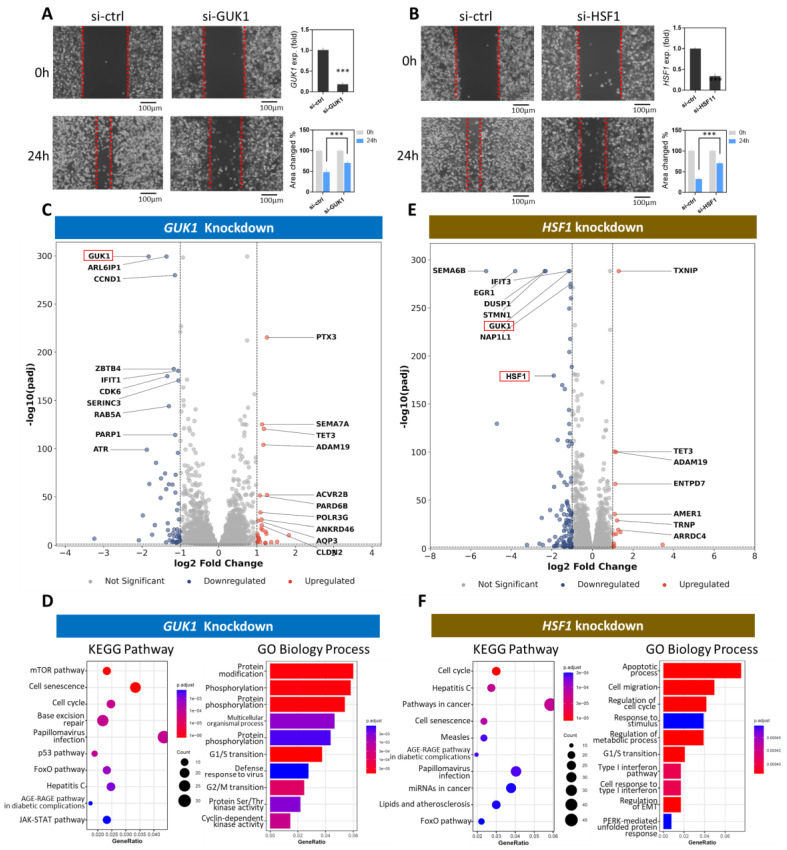
**Functional validation of the *HSF1-GUK1* axis in regulating breast cancer migration and proliferative signaling.** (**A**) Representative images of wound healing assays showing cell migration in control (si-ctrl) and *GUK1*-knockdown (si-GUK1) MDA-MB-231 breast cancer cells at 0 h and 24 h. Bar graphs (right) quantify *GUK1* knockdown efficiency by quantitative Polymerase Chain Reaction (qPCR) and the percentage of wound area closure. Data are presented as mean ± SD (***p < 0.001). (**B**) Wound healing assays performed on control (si-ctrl) and *HSF1*-knockdown (si-HSF1) cells. Bar graphs show *HSF1* knockdown efficiency and migration quantification. Data are presented as mean ± SD (***p < 0.001). (**C**,**D**) Volcano plot visualizing differentially expressed genes (DEGs) following *GUK1* knockdown (**C**) and HSF1 knockdown (**D**). (**E**) Dot plots showing KEGG pathway (left) and GO biological process (right) enrichment analysis for genes regulated by *GUK1*. The size of the dots represents gene count, and color indicates adjusted *p*-value. (**F**) KEGG and GO enrichment analysis for genes regulated by *HSF1*, showing overlap in cell cycle and signaling pathways with *GUK1* targets.

To validate the oncogenic functions of *GUK1* and its regulation by *HSF1 in vitro*, we performed siRNA-mediated knockdown assays in breast cancer cells. Scratch wound healing assays demonstrated that silencing *GUK1* significantly impaired cell migration compared to controls, with the wound gap remaining notably wider at 24 h (Fig. 6A). Importantly, knockdown of *HSF1* recapitulated this phenotype, leading to a comparable reduction in migratory capacity (Fig. 6B), which supports the hypothesis that these two factors operate within the same functional axis.

We next explored the downstream transcriptomic consequences of *GUK1* depletion. Differential expression analysis revealed that *GUK1* knockdown led to the significant downregulation of critical cell cycle regulators, including *CCND1* (Cyclin D1) and *CDK6*, as well as DNA repair genes like *PARP1* (Fig. 6C). Conversely, stress response and inflammatory markers such as *PTX3* were upregulated. Functional enrichment analysis confirmed that *GUK1* depletion suppressed pathways essential for tumor progression, specifically the mTOR pathway, Cell cycle, and p53 pathway (Fig. 6D). Furthermore, we observed a significant negative enrichment of the Ectonucleotidases (The Adenosine Pathway) gene set (Supplementary Fig. S3). Specifically, *GUK1* knockdown resulted in a Normalized Enrichment Score (NES) of −1.618 (*p* = 0.006), indicating that *GUK1* is essential for maintaining the expression of key enzymes in the adenosine signaling axis. These results suggest that the phenotypic consequences of *GUK1* depletion are, at least in part, mediated by the downregulation of adenosine metabolism. Finally, to mechanistically confirm the regulatory hierarchy, we analyzed the transcriptomic shifts following *HSF1* knockdown. Strikingly, *GUK1* was identified as one of the most significantly downregulated genes upon *HSF1* knockdown (Fig. 6E), providing direct transcriptomic evidence that *HSF1* drives *GUK1* expression. Furthermore, *HSF1* knockdown suppressed gene signatures overlapping with those of *GUK1*, including “Cell cycle” and “FoxO signaling”, while inducing apoptotic processes (Fig. 6F). Collectively, these data substantiate a mechanism where the *TP53-HSF1* axis upregulates *GUK1* to fuel metabolic and proliferative signaling via the mTOR and cell cycle pathways.

### 3.7 GUK1 Upregulation Activates the PI3K-AKT-mTOR Signaling Axis and Modulates Therapeutic Sensitivity

**Figure 7:**
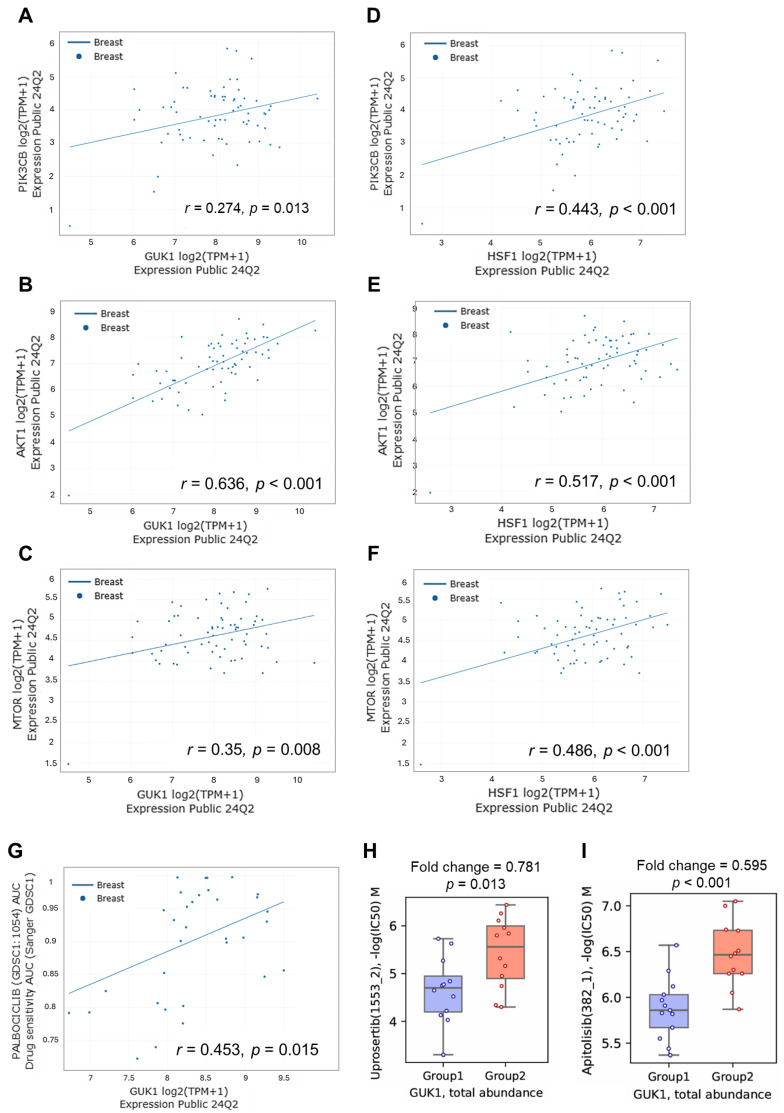
*GUK1* and *HSF1* expressions correlate with PI3K-AKT-mTOR pathway components and modulate therapeutic sensitivity in breast cancer. (**A**–**F**) Scatter plots derived from DepMap (Public 24Q2) illustrating Pearson correlations between mRNA expression levels. (**A**–**C**) *GUK1* expression is significantly positively correlated with (**A**) Phosphatidylinositol-4,5-bisphosphate 3-kinase catalytic subunit beta isoform (*PIK3CB*), (**B**) AKT serine/threonine kinase 1 (*AKT1*), and (**C**) mechanistic target of rapamycin kinase (*MTOR*). (**D**–**F**) Similarly, *HSF1* expression shows significant positive correlations with (**D**) *PIK3CB*, (**E**) *AKT1*, and (**F**) *MTOR*. (**G**) Correlation analysis between *GUK1* expression and drug sensitivity Area Under Curve (AUC) values for the CDK4/6 inhibitor Palbociclib (Sanger GDSC1 dataset). (**H**,**I**) Box plots comparing drug sensitivity (measured as −log(IC_50_), where IC_50_ stands for half maximal inhibitory concentration) between groups with low versus high *GUK1* total protein abundance. High *GUK1* abundance is associated with significantly increased sensitivity to (**H**) the Akt inhibitor Uprosertib and (**I**) the PI3K inhibitor Apitolisib. Statistical significance: Pearson’s correlation coefficients (*r*) and *p*-values are indicated in individual panels.

To further elucidate the functional consequences of the *HSF1*-*GUK1* axis beyond proliferation, we investigated its integration with oncogenic signaling networks. Analysis of the Cancer Dependency Map (DepMap) revealed that *GUK1* expression in breast cancer cell lines is significantly positively correlated with key components of the PI3K-AKT-mTOR pathway, including the catalytic subunit *PIK3CB* (*r* = 0.274, *p* = 0.013; Fig. 7A), the signal transducer *AKT1* (*r* = 0.636, *p* < 0.001; Fig. 7B), and the downstream effector *MTOR* (*r* = 0.35, *p* = 0.008; Fig. 7C). A similar and even stronger pattern of co-expression was observed for the upstream regulator *HSF1* (Fig. 7D–F), suggesting that the *TP53*-*HSF1*-*GUK1* axis functions as a coordinated module to sustain PI3K signaling.

To directly validate whether these transcriptomic correlations translate to functional pathway activation *in vitro*, we assessed the phosphorylation status of key downstream signaling molecules. Western blot analysis of MDA-MB-231 cells demonstrated that siRNA-mediated silencing of *GUK1* significantly attenuated the phosphorylation of AKT at Ser473 and mTOR at Ser2448, without significantly altering the total protein levels of AKT or mTOR (Supplementary Fig. S4A). Quantitative densitometry confirmed a statistically significant reduction in both the p-AKT/AKT and p-mTOR/mTOR ratios following *GUK1* knockdown (Supplementary Fig. S4B). These direct molecular readouts substantiate our *in silico* findings, definitively confirming that *GUK1* is required to maintain the active state of the PI3K-AKT-mTOR signaling cascade.

We next evaluated whether this pathway activation influences therapeutic response. While *GUK1* expressions play a role in cell cycle pathway (as shown in Fig. 5 and Fig. 6), its overexpression appears to confer resistance to standard cell cycle blockade; we observed a positive correlation between *GUK1* expression and the Area Under the Curve (AUC) values for the CDK4/6 inhibitor Palbociclib (*r* = 0.453, *p* = 0.015; Fig. 7G), indicating that tumors with high *GUK1* levels are less sensitive to this agent.

In contrast, high *GUK1* protein abundance was significantly associated with increased sensitivity to agents targeting the PI3K pathway. Breast cancer cell lines with high *GUK1* levels (red bar) exhibited significantly lower IC50 values (represented as higher −log(IC50)) for the AKT inhibitor Uprosertib (Fig. 7H) and the dual PI3K/mTOR inhibitor Apitolisib (Fig. 7I). These findings suggest that *GUK1* upregulation creates a specific dependency on the PI3K-AKT-mTOR axis, identifying a subset of patients who may benefit more from PI3K-targeted therapies than from standard CDK4/6 inhibition.

### 3.8 GUK1 Modulates Sensitivity to Apitolisib through Direct Physical Interaction

**Figure 8:**
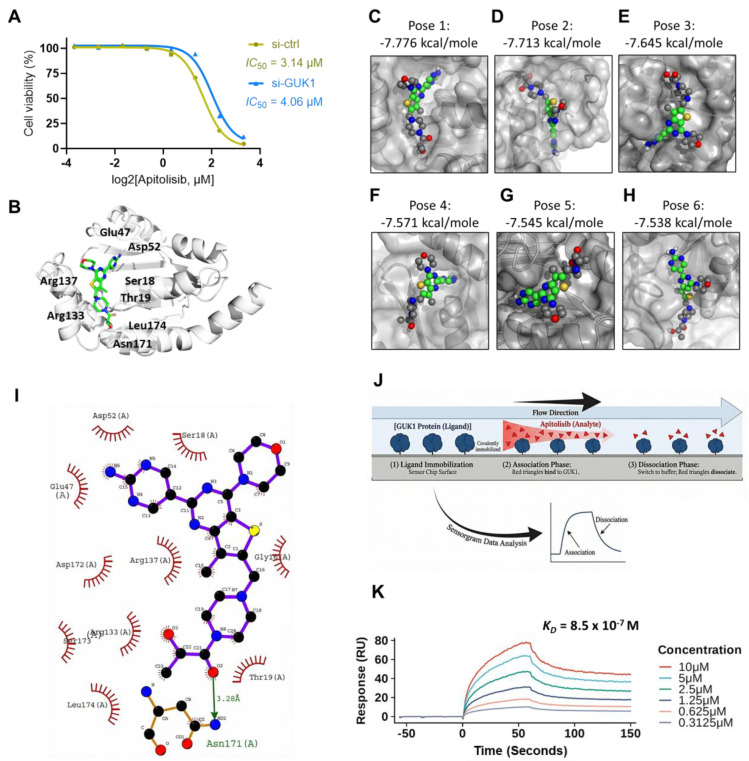
***GUK1* determines Apitolisib sensitivity and exhibits direct high-affinity binding.** (**A**) Cell viability curves of control (si-ctrl) and *GUK1*-knockdown (si-GUK1) breast cancer cells treated with increasing concentrations of Apitolisib. The *IC*_50_ values indicate that *GUK1* depletion confers resistance (increases *IC*_50_ from 3.14 μM to 4.06 μM). (**B**) 3D structural model of the GUK1-Apitolisib complex generated via AlphaFold and PyRx, visualizing the binding pocket and key surrounding residues. (**C**–**H**) Visualization of six distinct docking poses (Pose 1–6) for Apitolisib within the GUK1 active site. The calculated binding affinity energies (kcal/mol) are indicated above each panel, demonstrating consistently favorable interaction potentials. (**I**) 2D LigPlot interaction map of the most stable docking conformation (Pose 1). The diagram highlights a hydrogen bond (green dashed line) with residue Asn171 (3.28 Å) and hydrophobic contacts (red spoked arcs) with residues including Asp52, Ser18, and Glu47. (**J**) Schematic representation of the Surface Plasmon Resonance (SPR) assay setup, showing the immobilization of GUK1 protein (Ligand) and the flow of Apitolisib (Analyte) across the sensor chip surface. (**K**) Surface Plasmon Resonance (SPR) sensorgrams showing the real-time binding kinetics of Apitolisib to *GUK1* at various concentrations (0.3125 μM to 10 μM). The equilibrium dissociation constant (*K_D_*) is calculated at 8.5 × 10^−7^ M.

To further clarify the detailed interaction mechanism underlying the observed sensitivity to Apitolisib, we investigated whether *GUK1* expression influences the efficacy of Apitolisib. Cell viability assays demonstrated that siRNA-mediated knockdown of *GUK1* significantly reduced sensitivity to Apitolisib. The half-maximal inhibitory concentration (*IC*_50_) shifted from 3.14 μM in control cells to 4.06 μM in *GUK1*-silenced cells (Fig. 8A), confirming that high *GUK1* levels enhance drug sensitivity.

To elucidate the molecular basis of this interaction, we performed an integrated structural analysis. Molecular docking simulations using AlphaFold and PyRx predicted that Apitolisib fits snugly within the GUK1 binding pocket (Fig. 8B). We identified six distinct docking conformations with highly favorable binding energies ranging from −7.776 to −7.538 kcal/mol, suggesting a thermodynamically stable complex (Fig. 8C–H). Ligand-interaction mapping of the most stable pose revealed that the complex is anchored by a critical hydrogen bond (3.28 Å) between the side chain of Asn171 and Apitolisib, reinforced by a network of hydrophobic interactions involving residues Asp52, Ser18, and Glu47 (Fig. 8I).

To definitively validate this direct interaction *in vitro*, we employed Surface Plasmon Resonance (SPR) assays. Recombinant *GUK1* protein was immobilized on the sensor chip, and varying concentrations of Apitolisib were injected as the analyte (Fig. 8J). The resulting sensorgrams displayed dose-dependent binding responses with rapid association and dissociation kinetics (Fig. 8K). The calculated equilibrium dissociation constant (*K_D_*) was 8.5 × 10^−7^ M, confirming that Apitolisib binds *GUK1* with high affinity. Collectively, these data indicate that *GUK1* is not only a downstream effector of the TP53-HSF1 axis but also a direct physical target of dual PI3K/mTOR inhibitor Apitolisib, potentially explaining the enhanced therapeutic response in *GUK1*-high tumors.

## 4 Discussion

This study identifies *GUK1* as a pivotal metabolic driver and prognostic biomarker in breast cancer, significantly associated with advanced disease stages and poor survival outcomes. By integrating spatial transcriptomics, we demonstrated that *GUK1* colocalizes with expanding cancer cell nests, reinforcing its role in tumor progression. Mechanistically, we elucidated a novel TP53-HSF1-GUK1 axis, where *TP53* mutations strongly correlate with *HSF1* promoter hypomethylation and the downstream upregulation of *GUK1*. Beyond metabolism, *GUK1* expression correlates with an immunosuppressive microenvironment. Crucially, we provided direct *in vitro* molecular validation demonstrating that *GUK1* is required for the phosphorylation and activation of the PI3K-AKT-mTOR signaling cascade. Notably, while high *GUK1* levels predict resistance to CDK4/6 inhibitors, they enhance sensitivity to the dual PI3K/mTOR inhibitor Apitolisib through a direct, high-affinity physical interaction. These findings position *GUK1* as a robust candidate for precision oncology, offering a strategic target for overcoming therapeutic resistance in invasive breast cancer.

Further analysis emphasizes the relationship between *GUK1* and immune cell profiles within the TME. Notably, higher levels of *GUK1* correlate with reduced infiltration of endothelial cells, macrophages, and CD8^+^ T cells. In this context, endothelial cells are viewed as indicators of angiogenesis; reduced angiogenesis leads to localized hypoxia in tumor tissues. This low-oxygen environment fosters anaerobic metabolism, resulting in high lactate production, which may inhibit T cell activity [37]. Our results revealed a positive correlation between *GUK1* overexpression and the presence of exhausted T cells, indicating a TME that may hinder effective anti-tumor responses. This notion can be supported by the positive association of *GUK1* with immunosuppressive markers like *CTLA4* and *PDCD1* [38], as well as negative correlation with immunostimulatory markers such as CD28 and CD80 [39].

Previous studies have shown that abnormal p53 protein may affect its binding with DNA methyltransferase enzymes, leading to a failure in maintaining the methylation status of specific genes, thereby causing their overexpression [40,41]. The *TP53* gene mutations also potentially contributes to the accumulation of DNA mutations in the cancer cells, further driving gene amplification or deletion [42]. Therefore, our findings suggest that the increased expression of *HSF1* in the presence of *TP53* mutations may arise from two possible reasons. Firstly, it may result from the dysfunction of DNA methyltransferase enzymes, leading to hypomethylation of the promoter region of *HSF1*. Secondly, it may be due to the mutation of the tumor suppressor gene *TP53* indirectly causing amplification of the *HSF1* gene, thereby increasing its expression. This hypothesis is supported by our research results, where the expression levels of *HSF1* and *GUK1* are positively correlated with DNA damage pathway activity [43]. However, we acknowledge that the mechanistic evidence supporting this hypothesis is mainly derived from integrated bioinformatic analyses without direct experimental validation. This represents a significant limitation, and further experimental studies are required to clarify the regulatory relationship among *TP53*, *HSF1*, and *GUK1*.

Additionally, we found that the expression of *HSF1* and *GUK1* positively correlate with cell cycle activity in BC tumor cells, directly linking to cell cycle-related pathways. Our analysis showed that these genes also positively associate with *CDK4*, *CCND3*, and the PI3K-AKT-mTOR pathway [44]. Based on these findings, we further analyzed the relationship between GUK1 expression and the sensitivity of BC cell lines to targeted therapy drugs. Drugs analyzed included Palbociclib [45], a CDK4/6 inhibitor commonly used as a first-line treatment for breast cancer, and other targeted drugs like Uprosertib [46] and Apitolisib [47], which are not yet applied in breast cancer treatment. The results indicated that high *GUK1* expression enhances the sensitivity of cancer cells to these drugs. Despite these findings, due to the lack of experimental validation using drug-response assays in breast cancer cell lines stratified by *GUK1* expression, further *in vitro* studies are required to confirm the real drug effects in BC patients. Although drugs targeting the PI3K-AKT-mTOR pathway are not routinely used in breast cancer treatment, our findings suggest that high *GUK1* expression may serve as a potential marker for the future therapeutic use of these drugs in treating breast cancer.

While previous studies have utilized genomic screens to identify drug sensitivities, our study validates these findings through structural modeling. We demonstrated that the enhanced sensitivity of *GUK1*-overexpressing cells to Apitolisib is likely driven by a direct molecular interaction. Using AlphaFold-generated models, we identified a high-affinity binding pocket where Apitolisib forms a critical hydrogen bond with Asn171. This primary interaction is stabilized by a dense hydrophobic cage comprising residues such as Asp52, Ser18, and Arg133, ensuring the drug is retained within the protein core. These structural insights argue that *GUK1* expression is not just a passive biomarker for PI3K pathway activation but a potential direct target for Apitolisib, supporting its utility in precision oncology for invasive breast cancer.

The clinical implications of the *TP53*-*HSF1*-*GUK1* axis are particularly profound for aggressive breast cancer subtypes. Our data reveals that this regulatory axis is most active in TNBC and HER2-positive tumors (Fig. 2F), subtypes notoriously characterized by a high frequency of *TP53* mutations [48,49,50,51,52]. This suggests that the *TP53*-*HSF1*-*GUK1* signature may serve as a critical stratifying biomarker. While *TP53* mutations themselves have historically been difficult to target directly, our findings indicate that *GUK1* represents a druggable downstream metabolic effector. Consequently, patients with *TP53*-mutant TNBC—who currently face limited targeted therapy options—may represent the ideal cohort for *GUK1*-directed interventions, specifically using dual PI3K/mTOR inhibitors like Apitolisib to exploit the axis-dependent metabolic vulnerability.

Furthermore, our single-cell profiling highlights a potential role for *GUK1* in orchestrating an immunosuppressive microenvironment. The significant correlation between GUK1 overexpression and markers of T-cell exhaustion (*PDCD1*, *CTLA4*, *LAG3*) (Fig. 3) suggests that *GUK1*-driven metabolic reprogramming may contribute to immune evasion. High nucleotide metabolism in cancer cells often depletes the microenvironment of essential metabolites required for T-cell effector function. Notably, we observed that *GUK1* expression tracked significantly with the immunosuppressive cytokine *TGFB1* and key components of the adenosine signaling pathway, *ENTPD1* (CD39) and *NT5E* (CD73). We postulate that this coordinate upregulation fosters an adenosine-rich milieu that directly contributes to the observed CD8^+^ T-cell exhaustion, thereby limiting the efficacy of anti-tumor immunity.

To translate these mechanistic insights into clinical reality, rigorous *in vivo* validation is the necessary next step. Future studies will focus on the construction of genetically engineered mouse models (GEMMs), including conditional *Guk1* knockout (*Guk1^fl^*^/*fl*^) and *GUK1* transgenic strains, crossed with *Tp53*-mutant backgrounds. These models will be instrumental in verifying the oncogenic dependency on *GUK1* in a spontaneous tumor setting and, crucially, for evaluating the therapeutic efficacy of Apitolisib—both as a monotherapy and in combination with ICIs—assessing its ability to curb metastasis and extend survival in a living organism.

Despite the comprehensive multi-omics and functional insights provided by this study, several limitations must be acknowledged. First, while we successfully established a downstream regulatory link where *HSF1* knockdown leads to a significant reduction in *GUK1* expression, the upstream initiation of this axis via *TP53* mutation-mediated *HSF1* promoter hypomethylation remains primarily supported by integrated bioinformatic analyses. Although our clinical data clearly show a significant correlation between *TP53* status and *HSF1* methylation levels, direct experimental validation using DNA methyltransferase inhibitors, such as 5-Aza-2′-deoxycytidine (5-Aza-CdR), is required to definitively confirm the epigenetic priming mechanism. Furthermore, while Visium HD spatial transcriptomics provided high-resolution evidence of *GUK1* colocalization with expanding cancer cell nests, these observations were limited by a relatively small sample size. Future studies utilizing larger, independent spatial cohorts are necessary to fully assess the heterogeneity of *GUK1* expression across diverse breast cancer subtypes and to validate its spatial dynamics throughout all stages of disease progression. Finally, although our structural modeling and Surface Plasmon Resonance (SPR) assays demonstrate high-affinity binding between GUK1 and Apitolisib, future *in vivo* studies and prospective clinical trials are necessary to validate the therapeutic efficacy of targeting this metabolic vulnerability in patients with *TP53*-mutant breast cancer.

To bridge the gap between our preclinical findings and clinical application, we propose a stepwise framework to establish *GUK1* as a companion diagnostic for PI3K/mTOR inhibitor therapy. First, the immediate priority is assay standardization. A clinical-grade immunohistochemistry (IHC) protocol must be developed and validated according to CLIA standards, establishing precise H-score cut-offs that define ‘*GUK1*-high’ status specifically within *TP53*-mutant and TNBC populations. Second, retrospective clinical validation should be conducted using archival tumor samples from previous trials involving PI3K/mTOR inhibitors. This analysis aims to confirm whether high GUK1 protein expressions statistically correlate with improved objective response rates (ORR) or progression-free survival (PFS) in patients who received these agents. Finally, the definitive step would be a prospective biomarker-driven trial. We envision a Phase II basket trial enrolling patients with refractory, *TP53*-mutant metastatic breast cancer. Patients would be stratified based on *GUK1* status prior to treatment with Apitolisib (or next-generation dual inhibitors). Such a trial would directly test the hypothesis that GUK1 serves as a predictive biomarker for therapeutic sensitivity, potentially establishing a new precision oncology paradigm for these difficult-to-treat subtypes.

## 5 Conclusion

**Figure 9:**
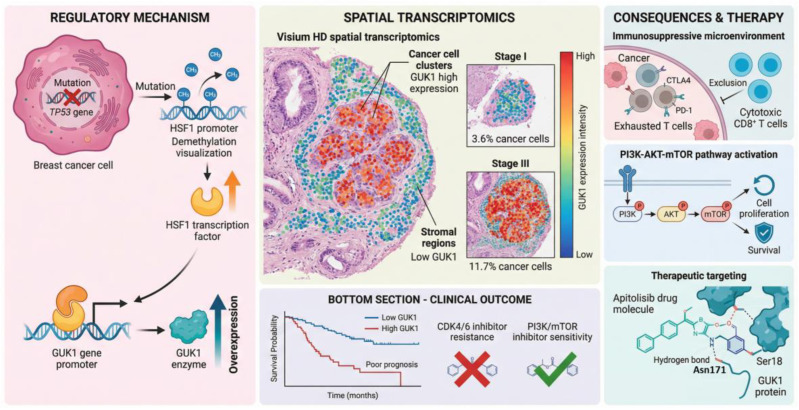
**Schematic summary of the *TP53*-*HSF1*-*GUK1* axis as a prognostic biomarker and therapeutic target in breast cancer.** *Regulatory Mechanism: TP53* mutations are closely associated with *HSF1* promoter hypomethylation, leading to the accumulation of the HSF1 transcription factor and the subsequent overexpression of the *GUK1* enzyme. *Spatial Transcriptomics:* Visium HD analysis demonstrates that *GUK1* expression (red/orange) is not ubiquitous but strongly colocalizes with expanding cancer cell clusters, intensifying from Stage I (3.6%) to Stage III (11.7%). *Consequences & Therapy: GUK1* upregulation fosters an immunosuppressive microenvironment enriched with exhausted T cells (expressing *CTLA4* and *PD-1*) and excludes cytotoxic CD8^+^ T cells. Mechanistically, this axis activates the PI3K-AKT-mTOR pathway, promoting cell proliferation and survival. Clinically, high *GUK1* levels correlate with poor survival and resistance to CDK4/6 inhibitors but predict sensitivity to PI3K/mTOR inhibitors. **Therapeutic Targeting:** Molecular docking confirms Apitolisib binds directly to the GUK1 protein pocket with high affinity (−7.7 kcal/mol), stabilized by a hydrogen bond at Asn171.

Our study establishes *GUK1* as a pivotal prognostic biomarker in breast cancer, driven by a novel *TP53*-*HSF1* epigenetic axis, as summarized in the schematic model presented in Fig. 9. High-definition spatial transcriptomics reveals *GUK1* specifically localizes to expanding cancer nests and fosters an immunosuppressive microenvironment. Mechanistically, this axis activates PI3K-AKT-mTOR signaling, creating a specific metabolic vulnerability. Crucially, we demonstrated that high *GUK1* levels, while predicting resistance to CDK4/6 inhibitors, confer significant sensitivity to the dual PI3K/mTOR inhibitor Apitolisib. This therapeutic link is not merely correlative; we confirmed via Surface Plasmon Resonance (SPR) and functional knockdown assays that *GUK1* is a direct physical target of Apitolisib and functionally determines drug response. Consequently, *GUK1* represents a promising precision oncology target, guiding therapeutic selection for patients with aggressive, *TP53*-mutant disease.

## Data Availability

The data that support the findings of this study are available from the Corresponding Author, upon reasonable request.

## Supplementary Materials

The supplementary material is available online at https://www.techscience.com/doi/10.32604/or.2026.078813/s1.
