# Supplementary material for: Integrated Multi-Omics and Spatial Transcriptomics Reveal GUK1 as a Prognostic Biomarker Regulated by the TP53-HSF1 Axis in Breast Cancer
Source: Oncol Res. 2026 Jul 16;34(8):16. doi: 10.32604/or.2026.078813 (PMC13397350; doi:10.32604/or.2026.078813)
Supplement: Supplementary file 1 [file OncolRes-34-78813-s001.zip › TSP_OR_78813-s001.docx]

**Supplementary Materials**

**
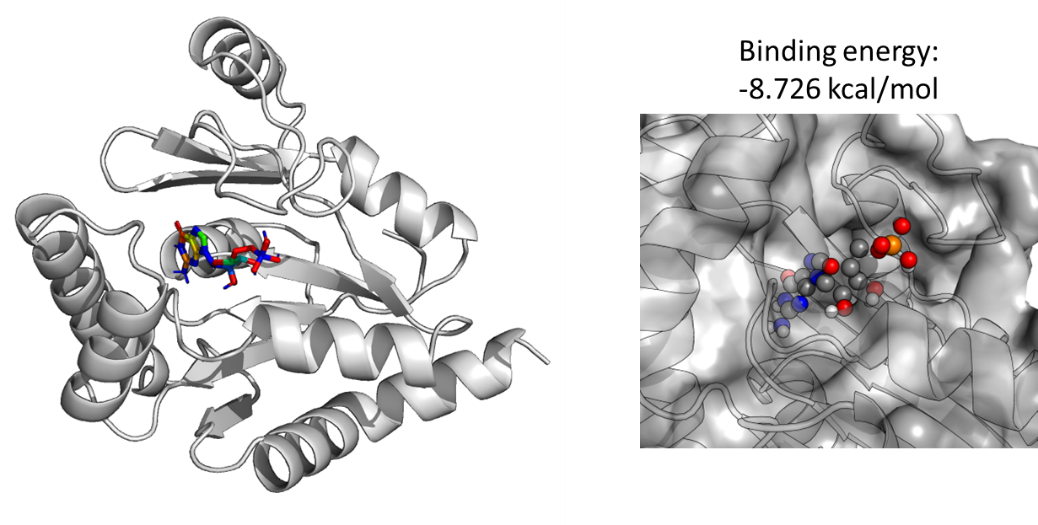
**

**Supplementary Figure S1. Computational validation of the molecular docking protocol.** The known endogenous substrate, guanosine monophosphate (GMP), was docked into the predicted active site of the human GUK1 structural model using the AutoDock Vina algorithm. The left panel displays the overall three-dimensional conformation of the GUK1-GMP complex (cartoon representation). The right panel provides a close-up surface view of the core nucleotide monophosphate (NMP) binding pocket, demonstrating a highly favorable binding affinity of -8.726 kcal/mol. This procedure empirically validates the accuracy of the grid box and system parameters applied in the subsequent screening for Apitolisib.

**
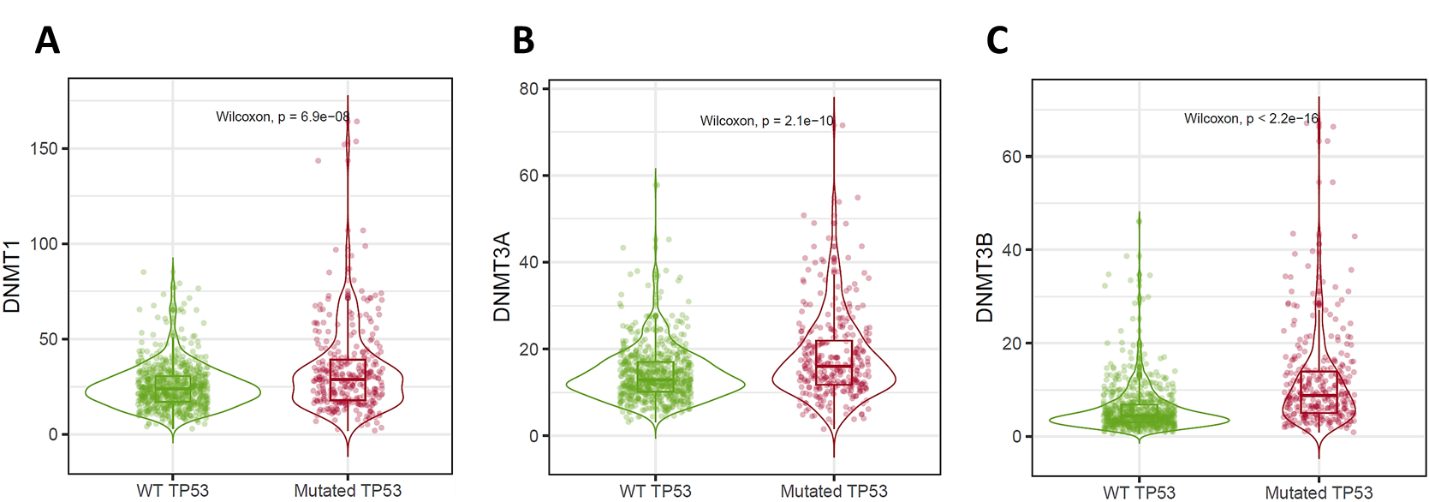
**

**Supplementary Figure S2. Correlation between *TP53* mutation status and DNA methyltransferase expression in TCGA-BRCA patients.** Violin plots illustrate mRNA expression levels of **(A)** DNMT1, **(B)** DNMT3A, and **(C)** DNMT3B in TCGA breast invasive carcinoma (BRCA) cohorts. Patients are stratified by *TP53* status: wild type (WT, green) versus mutated (red). Each plot includes an internal box plot representing median and interquartile ranges, with individual patient data points overlaid. Statistical significance was determined using the Wilcoxon rank-sum test.


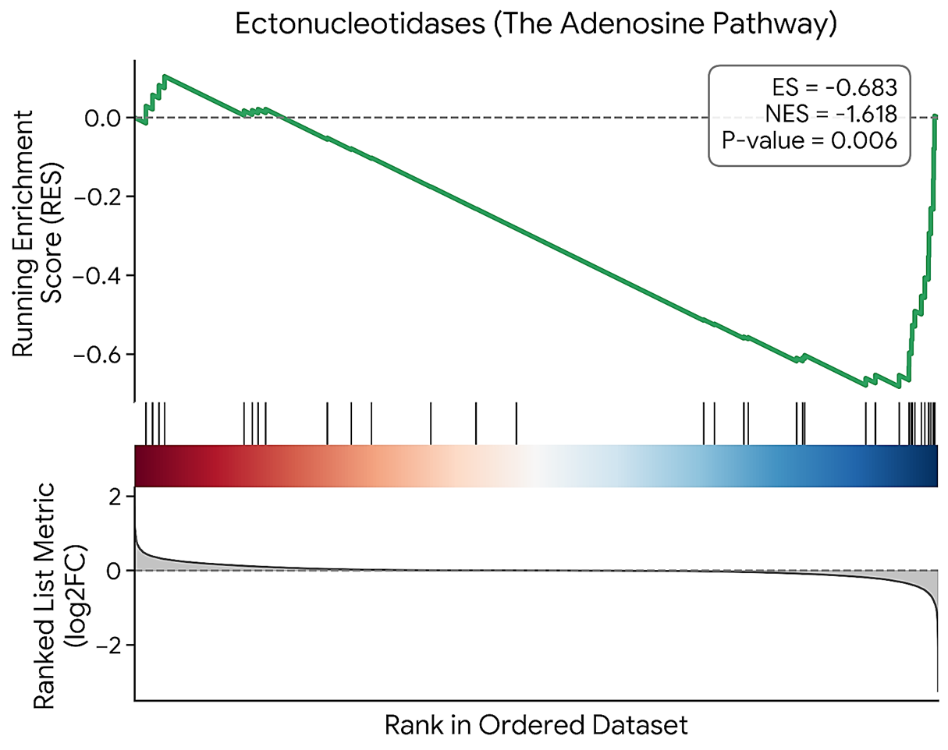


**Supplementary Figure S3. GSEA enrichment plot for the Adenosine Pathway (Ectonucleotidases) following *GUK1* knockdown.** Gene Set Enrichment Analysis (GSEA) was performed using RNA-seq data comparing si-ctrl and si-GUK1 treated cells. The enrichment plot for the "Ectonucleotidases (The Adenosine Pathway)" gene set shows a negative Enrichment Score (ES = -0.683) and a significant Normalized Enrichment Score (NES = -1.618, *p*-value = 0.006). The distribution of genes (vertical black bars) is skewed toward the right (blue) side of the rank-ordered list (downregulated in si-GUK1) confirms a significant transcriptional downregulation of this pathway upon *GUK1* silencing.


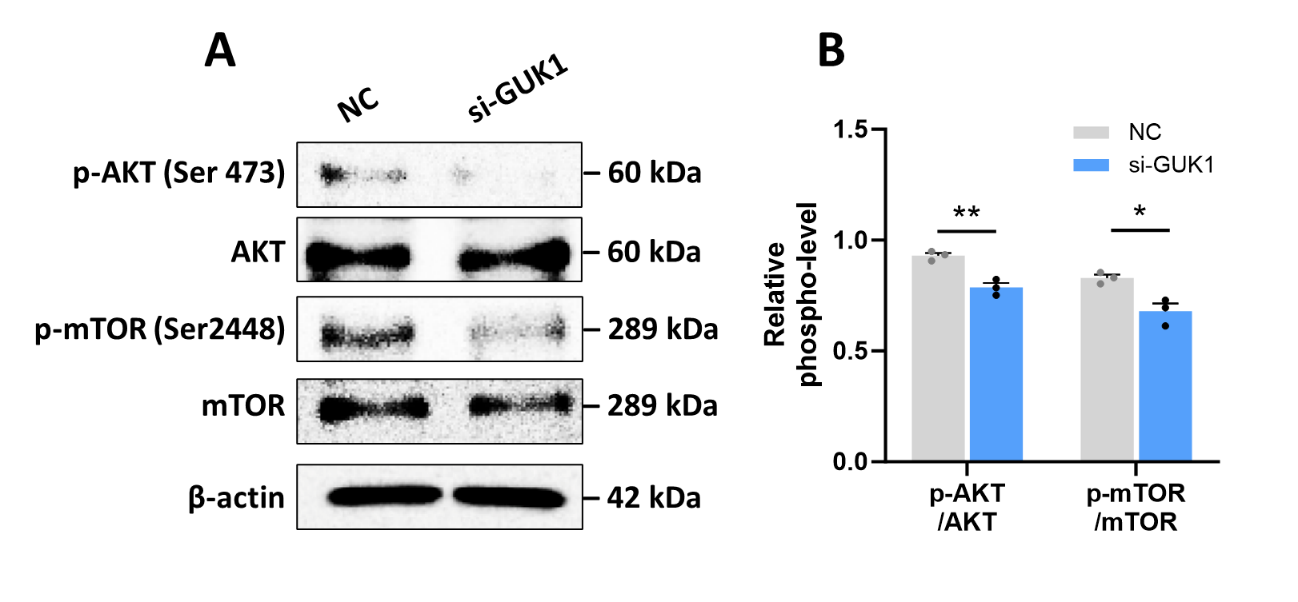


**Supplementary Figure S4. Knockdown of GUK1 attenuates AKT and mTOR phosphorylation. (A)** Representative Western blot analysis of phosphorylated AKT at Ser473 (p-AKT), total AKT, phosphorylated mTOR at Ser2448 (p-mTOR), and total mTOR in cells transfected with negative control siRNA (NC) or GUK1-specific siRNA (si-GUK1). β-actin was used as an internal loading control. **(B)** Quantitative densitometry analysis of relative p-AKT and p-mTOR levels. The phosphorylation levels were normalized to their respective total protein levels (p-AKT/AKT and p-mTOR/mTOR). Data are presented as mean ± SEM, n = 3 independent biological replicates. **p* < 0.05, ***p* < 0.01 by independent *t*-test.
